# Multi-cohort comprehensive analysis unveiling the clinical value and therapeutic effect of *GNAL* in glioma

**DOI:** 10.32604/or.2024.045769

**Published:** 2024-04-23

**Authors:** ZHEN LIU, LIANGWANG YANG, ZHENGXING XIE, HUI YU, TIANYI GU, DAOMING SHI, NING CAI, SHENGHUA ZHUO

**Affiliations:** 1 Department of Neurosurgery, Affiliated Hospital of Jiangsu University, Zhenjiang, 212000, China; 2 Department of Neurosurgery, First Affiliated Hospital of Hainan Medical University, Haikou, 570100, China; 3 Department of Cardiothoracic Surgery, Affiliated Hospital of Jiangsu University, Zhenjiang, 212000, China; 4 Department of General Surgery, Affiliated Hospital of Jiangsu University, Zhenjiang, 212000, China

**Keywords:** Glioma, Olfaction, *GNAL*, Tumor immune microenvironment, Immunotherapy/chemotherapy response

## Abstract

Clinical data indicates that glioma patients have poor treatment outcomes and clinical prognosis. The role of olfactory signaling pathway-related genes (OSPRGs) in glioma has not been fully elucidated. In this study, we aimed to investigate the role and relationship between OSPRGs and glioma. Univariate and multivariate Cox regression analyses were performed to assess the relationship between OSPRGs and the overall survival of glioma based on public cohorts, and the target gene (G Protein Subunit Alpha L, *GNAL*) was screened. The association of *GNAL* expression with clinicopathological characteristics, gene mutation landscape, tumor immune microenvironment (TIME), deoxyribonucleic acid (DNA) methylation, and naris-occlusion controlled genes (NOCGs) was performed. Immunohistochemistry was used to evaluate GNAL level in glioma. Further analysis was conducted to evaluate the drug sensitivity, immunotherapy response, and functional enrichment of *GNAL*. *GNAL* was an independent prognostic factor, and patients with low *GNAL* expression have a poor prognosis. Expression of *GNAL* was closely associated with clinicopathological characteristics, DNA methylation, and several immune-related pathways. Immune infiltration analysis indicated that *GNAL* levels were negatively correlated with immune scores. *GNAL* low-expression group showed efficacy with anti-PD-1 therapy. Ten compounds with significantly different half-maximal inhibitory concentration (IC50) values between the *GNAL* high and low-expression groups were identified. Furthermore, its expression was associated with several immune cells, immune-related genes, and NOCGs. The expression of *GNAL* is closely associated with clinicopathological characteristics, TIME, and the response to therapeutic interventions, highlighting its potential as a prognostic biomarker for glioma.

## Introduction

As the most aggressive and common malignant tumor of the central nervous system (CNS), glioma accounts for 24.5% of all primary brain tumors and other CNS tumors [1]. Adult-type diffuse gliomas were classified into World Health Organization (WHO) grades 2–4, each with varying degree of malignancy [2]. Specifically, glioblastoma (GBM), the WHO grade 4, is extremely malignant and aggressive and accounts for 49.1% of all CNS malignancies and the five-year survival rate for GBM patients is 6.8%, with a median survival rate of only eight months [1]. The existing research has demonstrated that individuals with *IDH* mutations and/or 1p/19q co-deletion exhibit a more favorable prognosis, while those with *IDH* wildtype or 1p/19q non-codeletion have a poor prognosis [3]. Additionally, *MGMT* promoter methylation can confer a beneficial effect by enhancing chemosensitivity with chemotherapy of alkylating agent, thereby ameliorating the prognosis [4]. Despite the many advances in glioma prevention, early detection, and prompt treatment, only a few significant advancements have been achieved [5]. New therapeutic methods, such as immunotherapy, molecular targeted therapy, gene therapy, and electric field therapy are also gradually being applied [6,7]. Due to its growth characteristics and specific tumor microenvironment (TME), these novel therapies have not improved the prognostic outcomes or quality of life for glioma patients. Administration of drugs for these complex tumors and achieving accurate treatment is a major clinical challenge [8].

The olfactory system is a crucial chemosensory system for detecting complex environmental cues, including odors of food and poisons [9]. When stimulated by external odors, olfactory receptors (ORs) expressed in olfactory sensory cells initiate a cascade of events, converting odorant-specific chemical information into electrical signals that relay olfactory stimuli to the brain [10]. It has been reported that nose-to-brain delivery is critical in regulating gliomas from the “cold” to the “hot” TME by bypassing the blood-brain barrier and directly delivering drugs to tumor sites [11,12]. This suggests that many physiological structures forming part of the olfactory system not only play a role in sensory information transmission but are also likely to be involved in important biological processes of CNS diseases.

The ORs are strongly correlated with tumor development. Activation of OR2J3 induces cell apoptosis, inhibits cell proliferation and migration, and therefore, suppresses the proliferation of non-small cell lung cancer cells [13]. Moreover, ORs exhibit certain functionality in the tumor cell inhibition process in colorectal cancer [14] and human myeloid leukemia [15]. These findings suggest that olfaction and its receptors play important roles in the development of multiple tumors. Interestingly, a recent study has shown that olfaction is closely related to the development and progression of glioma [16]. Chen et al. investigated the functions of *IGF1*, which exhibits consistent downregulation after naris occlusion in the olfactory bulb (OB). This study showed that olfaction directly modulated malignant glioma development by activating relevant olfactory neural circuits. However, the roles of olfactory signaling pathway-related genes (OSPRGs) in glioma have not been comprehensively analyzed.

To elucidate the relationship between OSPRGs and glioma, we used the sequencing data cohorts and systematically investigated the relationship between OSPRGs expression and survival rate by univariate and multivariate Cox regression analysis. The target gene *GNAL* was screened. *GNAL* can mediate signal transduction within the olfactory neuroepithelium [17]. Meanwhile, Kim et al. confirmed its mRNA expression is significantly higher in the olfactory training mouse group relative to the anosmia group [18].

In gliomas, *GNAL* has been identified as a hub-gene [19]. However, the expression, prognostic values, gene mutation landscape, functional enrichment, immunotherapeutic response, chemotherapeutic response, and the relationship between *GNAL* and tumor immune microenvironment (TIME) in glioma remain unclear. Herein, the impact of *GNAL* on glioma prognosis, its expressions in different clinicopathologic groups, gene mutation status, and functional enrichment were analyzed. The correlation between *GNAL* and TIME, immune-related genes, and naris-occlusion controlled genes (NOCGs) was also investigated, and DNA methylation levels were explored. Immunotherapy or chemotherapy responses were compared in different *GNAL* expression groups. These findings of this study provide a basis for future investigations into protective mechanisms of the *GNAL* gene in glioma.

## Materials and Methods

### Data acquisition

Public RNAseq data (fragments per kilobase of transcript per million mapped reads, FPKM; Chinese Glioma Genome Atlas (CGGA) mRNAseq_325 (CGGA325); CGGA mRNAseq_693 (CGGA693) as well as the Tumor Genome Atlas (TCGA) RNAseq (TCGA-GBMLG)) and the corresponding clinical (gender, age, overall survival (OS) and survival state) and pathology (WHO grade, *IDH* mutation status and 1p/19q codeletion status) data were obtained from CGGA (http://www.cgga.org.cn/index.jsp) within in-house and other data [20]. Recurring cases and those with deletion survival data in public RNA-Seq data were excluded. Primary tumors with complete survival data were selected for analysis. The detailed distribution of information for cohorts is shown in Suppl. Table S1. 417 OSPRGs (REACTOME_OLFACTORY_SIGNALING_PATHWAY) were obtained from the Molecular Signatures Database (MSigDB) (https://www.gsea-msigdb.org/gsea/index.jsp).

### Screening of target genes

29 OSPRGs to be studied were included after Venn map screening (Suppl. Table S2). Statistically significant shared genes were retained (*p* < 0.05) through univariate Cox regression analysis. The target gene (*GNAL*) was screened by multivariate Cox regression analysis. Cox regression analyses were performed using the Survival (3.4–0) package in R software (4.1.3), and hazard ratios (HR) along with their corresponding 95% confidence intervals (95% CI) were calculated.

### Survival analysis

Patients were assigned into high and low-expression groups based on median *GNAL* expression. Survival rates were determined by Kaplan-Meier (K-M) survival curve analysis. The K-M plots for CGGA325 and CGGA693 were completed on the CGGA website; the K-M plots for TCGA-GBMLGG, Rembrandt, and Gravendeel were developed using Gliovis (http://gliovis.bioinfo.cnio.es/) [21]; while the prognostic values of *GNAL* and clinicopathological characteristics were determined by multivariate Cox regression survival analysis.

### Differential analysis of GNAL mRNA expression and immunohistochemical staining

Differences in *GNAL* mRNA expression in different grades, genders, ages, *IDH* mutation status, 1p/19q codeletion status, and *IDH* mutation combined with 1p/19q codeletion status (*IDH*-1p/19q status) are shown in the box diagram. Expression differences in *GNAL* between different GBM subtypes (including classical, mesenchymal, and procedural) were analyzed in Gliovis. The GEPIA database (http://gepia.cancer-pku.cn/detail.php) [22] was used to analyze differences in *GNAL* mRNA expression in normal brain tissues compared with low-grade glioma (LGG) and GBM. Differences in GNAL protein levels between glioma and normal brain tissues were determined in the UALCAN database (http://ualcan.path.uab.edu/index.html) [23].

The expression of GNAL in glioma tissues was investigated using 101 glioma specimens, which were collected from the First Affiliated Hospital of Hainan Medical University. The studies involving human participants were reviewed and approved by the Humanities Ethics Committee of the First Affiliated Hospital of Hainan Medical University (Ethics Approval Number: 2023-KYL-124) and all research procedures were in accordance with the code of ethics of the Institution, the National Research Council, and with the 1975 Declaration of Helsinki and its subsequent amendments. And all participants were required to sign an informed consent prior to their inclusion in the study. The glioma diagnosis was established through pathological analysis of the tissue specimens by experts from the department of pathology, the First Affiliated Hospital of Hainan Medical University. 5 µm thick paraffin-embedded glioma tissue sections were blocked with 5% BSA (Sigma, B2064) for 20 min and incubated with primary polyclonal anti-*GNAL* (absin, abs141187, 1:200) overnight at 4°C. After being washed with phosphate buffered saline (PBS), the sections were incubated with biotinylated immunoglobulin G (IgG) (1:200) secondary antibodies for 30 min at 37°C. The secondary antibody and diaminobenzidine color development were executed utilizing the Dako REAL™ EnVision™ detection system. The stained sections’ scanned images were captured employing the digital pathology slide scanner (KFBIO KF-PRO-120). K-Viewer software (version 1.5.5.6) was utilized for graphical representation. The results were assessed by investigators, working independently, primarily based on the intensity of staining and the count of positive cells. The cell score of 0%–25% staining is (+, 1); cells with 25%–49% staining were scored as (++, 2); cells with 50%–74% staining were scored as (+++, 3); and cells with 75%–100% staining were scored as (++++, 4). The staining color was scored as light-yellow particle (+, 1), brown-yellow particle (++, 2), and brown particle (+++, 3). The final score was defined as staining number score multiplied by staining color score.

### Genetic alteration and functional enrichment analyses

The CBioPortal for Cancer Genomics database (https://www.cbioportal.org/) [24] was used to investigate *GNAL* genetic alteration characteristics. The “Mutation Landscape” module in the CAMOIP database (http://www.camoip.net/) [25] was used to further investigate gene mutations in patients with different GNAL mRNA expression in TCGA-GBM and TCGA-LGG.

To establish the biological functions and the pathways in which *GNAL* was enriched in glioma, gene ontology-biological process (GO-BP), gene ontology-cellular component (GO-CC), gene ontology-molecular function (GO-MF), Reactome pathway enrichment, and kyoto encyclopedia of genes and genomes (KEGG) pathway enrichment analyses were completed using “Gene set enrichment analysis (GSEA)” module under the “Pathway Enrichment” module in CAMOIP. The results of top 20 were visualized by Ridge-Plot.

### Immune infiltrations and immune-related gene analysis

To investigate the relationship between *GNAL* expression and TIME, Sangerbox bioinformatics analysis (http://sangerbox.com/home.html) [26] was performed to complete the immune infiltration correlation analysis. ImmueScore, StromalScore, and EstimateScore were evaluated using the ESTIMATE algorithm [27]. The EPIC algorithm was then used to explore the differences in immune cell expression between different *GNAL* expression groups [28]. According to the CIBERSORT algorithm [29], penetration levels of 22 types of tumor-infiltrating immune cells (TIICs) in the TIME of each sample were calculated using the deconvolution method, and differences in TIICs expression among different *GNAL* expression groups were compared. Moreover, the relationships between the expression of immunoinhibitors, immunostimulators, major histocompatibility complex (MHC) molecules, tumor-infiltrating lymphocytes, chemokines, and chemokine receptors in LGG and GBM, with *GNAL* were analyzed in TISIDB (http://cis.hku.hk/TISIDB/index.php) [30].

The list of immune-related genes was downloaded from the ImmPortPortal database (https://www.immport.org/home) [31]. Correlation analysis was performed to evaluate the association of *GNAL* expression with immune-related genes. Immune-related genes with Spearman correlation coefficient greater than 0.6 were retained and visualized with a correlation heatmap.

### Prediction of immunotherapy/chemotherapy response

The Submap algorithm [32] (https://cloud.genepattern.org/gp) was utilized to predict the clinical response to PD1 and CTLA4 immune checkpoint blocking within different *GNAL* expression groups. Pharmacogenomics database Genomics of Drug Sensitivity in Cancer (GDSC) was utilized to predict chemotherapeutic response [33]. The R package “pRRophetic” (version 0.5) was applied to achieve the prediction process, in which the samples’ IC50 was estimated by ridge regression.

### Analysis of gene methylation and NOCGs

The correlations between *GNAL* mRNA expression and promoter methylation levels in TCGA-LGG and TCGA-GBM were analyzed in the MEXPRESS database (https://www.mexpress.be/) [34]. To establish the association between olfaction and glioma [16], genes (*ADCYAP1*, *ALK*, *ANO3*, *APOLD1*, *ATF5*, *CCK*, *FOSL2*, *IGF1*, *NPTX2*, *OMP*, *PCSK1*, *SCG2*, *SYT10*, *TH*, *TRH*, and *ZNF804A*) which showed consistent downregulation after naris occlusion in OB and *GNAL* were analyzed.

### Statistical analysis

Wilcoxon’s rank sum test was utilized to compare differences in *GNAL* expression between and among groups. The Chi-square test was used to compare clinical data between patients in *GNAL* high and low-expression groups. The Log-rank test and K-M plot were used to compare the survival rates of *GNAL* high and low-expression groups. Univariate and multivariate Cox regression analyses were conducted to screen for the independent prognostic indicators. Spearman’s correlation coefficient was used for correlation analyses. *p* < 0.05 was the threshold for significance.

## Results

### Screening of target genes

**Figure 1:**
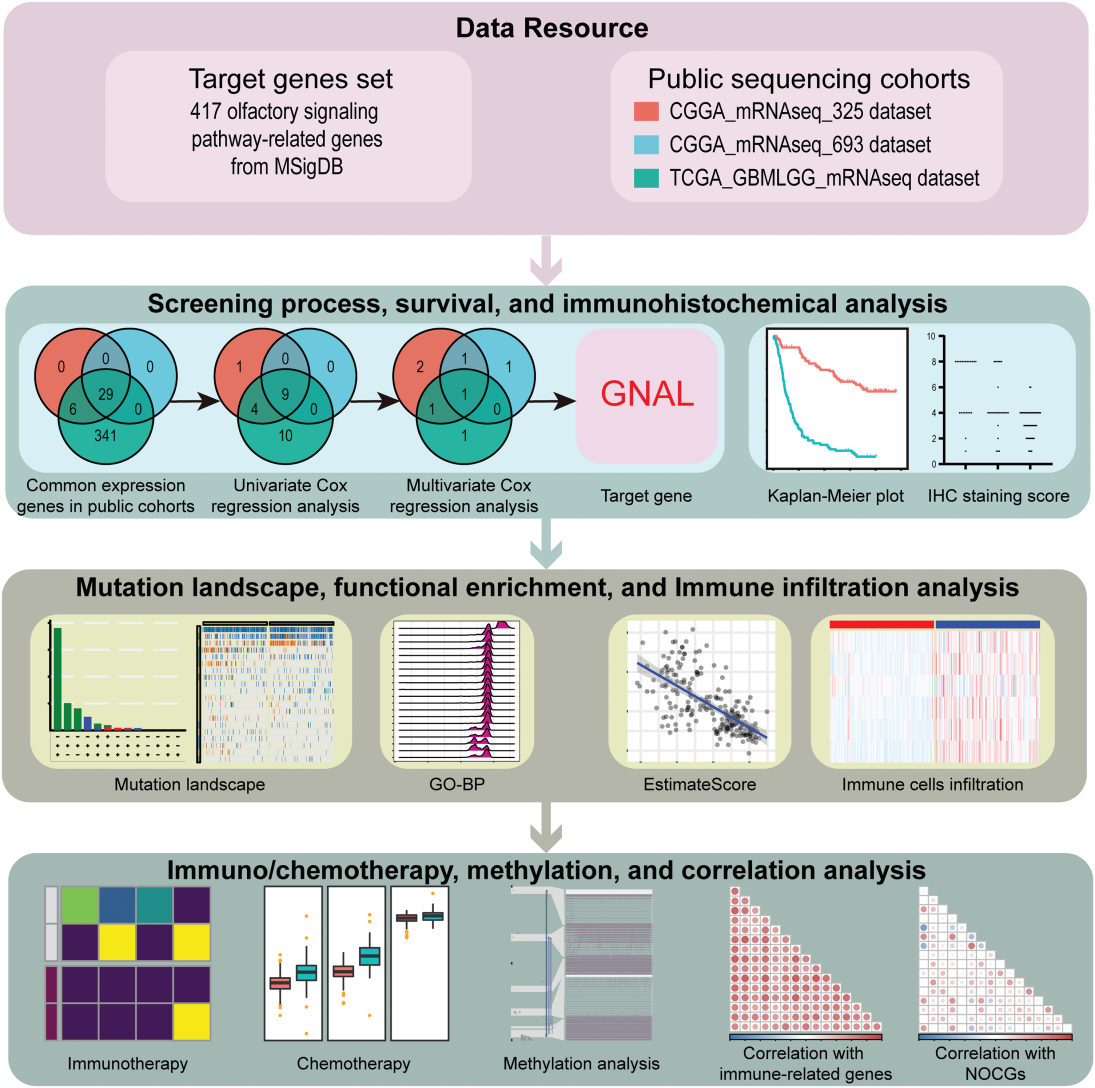
The flowchart of this study.

The flowchart for this study is shown in Fig. 1. Univariate Cox survival analysis was performed to determine the relationship between 29 OSPRGs and OS for glioma patients. In this study, 14, 9, and 23 genes associated with survival outcomes were screened from CGGA325, CGGA693, and TCGA-GBMLGG cohorts, respectively (Suppl. Fig. 1). Multivariate Cox regression survival analysis was performed on the genes with different survival rates to determine the genes that could independently influence the prognosis of patients. Finally, *GNAL*, was identified as the target gene (Table 1).

**Table 1:** Multivariate Cox regression analysis of OSPRGs associated with overall survival in public RNAseq cohorts

Dataset	Gene symbols	Coefficient	*p-*value	Hazard ratio (95% CI)
CGGA325	*ADCY3*	0.032	0.006	1.033 (1.009–1.057)
	*CNGB1*	0.277	0.038	1.319 (1.016–1.713)
	*GNAL*	−0.157	<0.001	0.855 (0.811–0.900)
	*GNB1*	0.004	0.030	1.004 (1.000–1.008)
	*GNG13*	−0.433	0.016	0.648 (0.455–0.923)
CGGA693	*GNAL*	−0.311	<0.001	0.733 (0.679–0.791)
	*GNB1*	0.005	0.002	1.005 (1.002–1.008)
	*REEP1*	0.055	0.017	1.056 (1.010–1.105)
TCGA-GBMLGG	*ADCY3*	0.758	<0.001	2.134 (1.465–3.109)
	*GNAL*	−0.314	<0.001	0.730 (0.656–0.813)
	*OR51E1*	0.178	<0.001	1.195 (1.087–1.314)

Note: 95% CI: 95% confidence intervals.

### Classification of glioma patients based on median GNAL expressions

The patients can be divided into high and low-expression groups according to their median *GNAL* expression, and the relationships between *GNAL* expression and the clinical parameters of patients were analyzed. In the CGGA325 cohort, the WHO grade (*p* < 0.001), age (*p* < 0.001), *IDH* mutation status (*p* < 0.001), 1p/19q codeletion status (*p* < 0.001), and the methylation status of MGMT promoter (*p* = 0.01) between high and low *GNAL* expression groups were significantly different (Table 2). The results showed that patients in the low *GNAL* expression group tended to have high WHO grade, old-age (≥42), *IDH* wildtype, and 1p/19q non-codeletion. And the above indicators are mostly indicators of poor prognosis for glioma patients [2], suggesting that decreased *GNAL* may be a prognostic indicator of glioma. Similar to the CGGA325 cohort, there were marked differences in WHO grade (*p* < 0.001), *IDH* mutation status (*p* < 0.001), and 1p/19q codeletion status (*p* < 0.001) between the groups with high and low *GNAL* expression in CGGA693 (Suppl. Table S3) and TCGA-GBMLGG cohorts (Suppl. Table S4).

**Table 2:** Characteristics of patients between *GNAL* high and low-expression groups in CGGA325 cohort

Characteristics	N	Low expression (N = 111)	High expression (N = 111)	*p-*value
Grade	222			<0.001
WHO II		14 (6.31%)	76 (34.23%)	
WHO III		23 (10.36%)	24 (10.81%)	
WHO IV		74 (33.33%)	11 (4.95%)	
Gender	222			0.68
Female		40 (18.02%)	44 (19.82%)	
Male		71 (31.98%)	67 (30.18%)	
Age	222			<0.001
<42		27 (12.16%)	67 (30.18%)	
≥42		84 (37.84%)	44 (19.82%)	
*IDH* mutation status	221			<0.001
Mutant		19 (8.60%)	93 (42.08%)	
Wildtype		92 (41.63%)	17 (7.69%)	
1p/19q codeletion status	219			<0.001
Codeletion		1 (0.46%)	49 (22.37%)	
Non-codeletion		109 (49.77%)	60 (27.40%)	
MGMTp methylation status	208			0.01
Methylated		39 (18.75%)	57 (27.40%)	
Un-methylated		66 (31.73%)	46 (22.12%)	

### GNAL is an independent prognostic indicator for glioma

**Figure 2:**
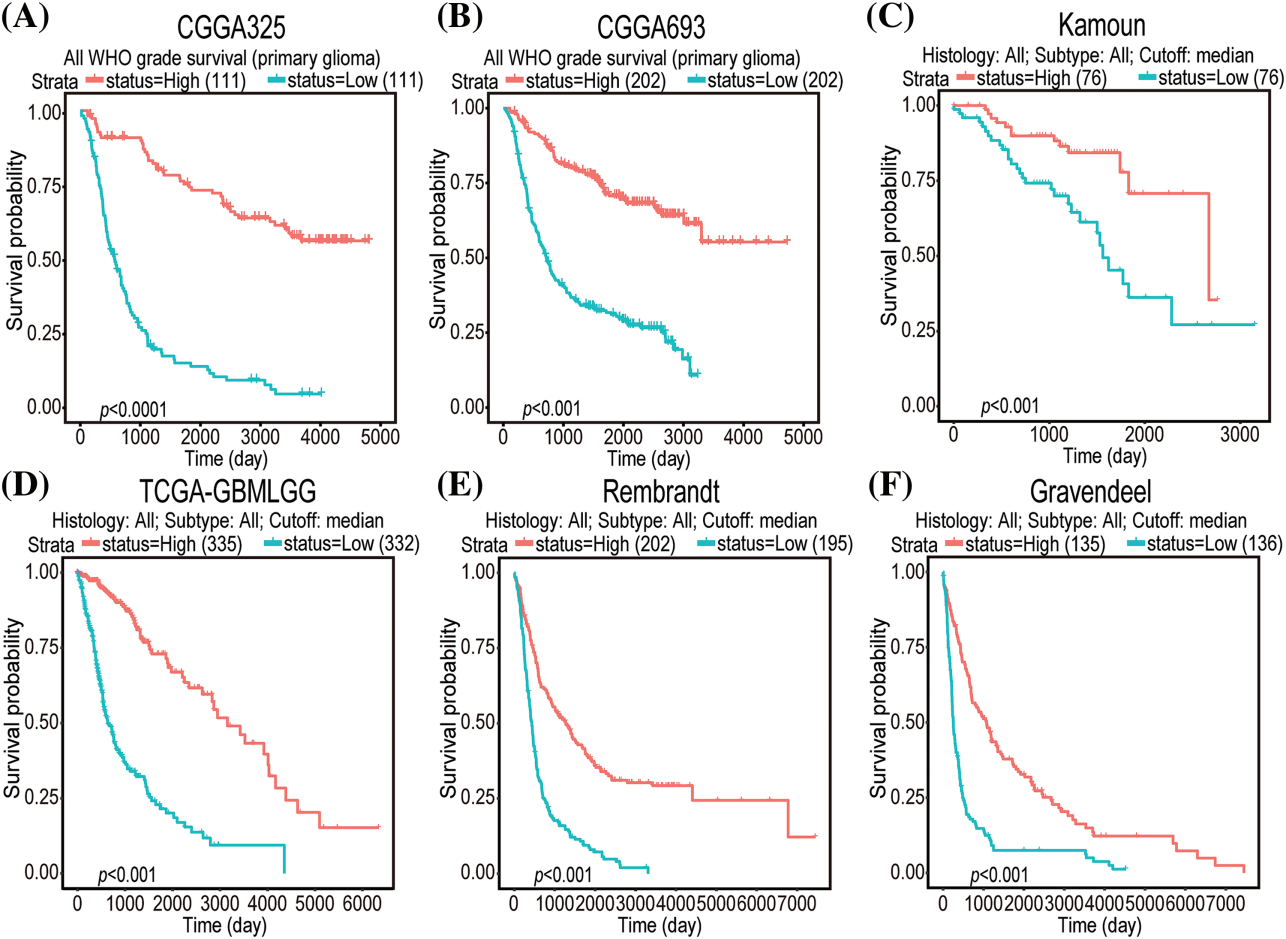
K-M survival curve plots of *GNAL* expressions in CGGA325 (A), CGGA693 (B), Kamoun (C), TCGA-GBMLGG (D), Rembrandt (E), and Gravendeel (F) cohorts.

Univariate Cox regression analysis of clinical factors and molecular characteristics in each cohort revealed that gender was not markedly associated with prognostic outcomes (Suppl. Table S5). To analyze the independent prognostic indicators, multivariate Cox regression analysis was performed to establish the prognostic values of *GNAL* expression, clinical factors, and molecular characteristics (Table 3). The inverse association between *GNAL* levels and survival outcomes was observed across all cohorts, providing compelling evidence that the *GNAL* expression status may be an independent prognostic biomarker. These findings imply that *GNAL* expressions are positively correlated with glioma prognosis. On the basis of this result, the predictive value of the *GNAL* for glioma prognosis was further analyzed through the K-M survival curve. The plots showed that gliomas with high *GNAL* expression have higher survival rates in CGGA325 and CGGA693 cohorts (Figs. 2A and 2B). The findings were validated in Kamoun, TGGA-GBMLGG, Rembrandt, and Gravendeel cohorts (Figs. 2C–2F). These results show that OS rates in the group with low *GNAL* expression are lower than those in the group with high *GNAL* expression, suggesting that low *GNAL* expression indicate poor prognosis in glioma.

**Table 3:** Multivariate Cox regression analysis of clinicopathologic characteristics and *GNAL* in public RNAseq cohorts

Dataset	Factors	Coefficient	*p-*value	Hazard ratio (95% CI)
CGGA325	Grade	0.682	0.002	1.978 (1.281–3.053)
	Age	0.029	<0.001	1.029 (1.013–1.046)
	1p/19q codeletion status	−1.371	<0.001	0.254 (0.118–0.546)
	MGMTp methylation status	−0.396	0.040	0.672 (0.460–0.983)
	*GNAL*	−0.901	<0.001	0.406 (0.244–0.676)
CGGA693	Grade	0.741	0.001	2.097 (1.331–3.305)
	Age	0.015	0.021	1.015 (1.002–1.028)
	*IDH* mutation status	−0.623	0.005	0.536 (0.347–0.828)
	1p/19q codeletion status	−0.910	0.013	0.403 (0.196–0.827)
	*GNAL*	−0.647	0.002	0.524 (0.347–0.790)
TCGA-GBMLGG	Grade	0.532	0.017	1.703 (1.099–2.638)
	Age	0.043	<0.001	1.043 (1.029–1.059)
	*IDH* mutation status	−1.248	<0.001	0.287 (0.171–0.484)
	*GNAL*	−0.562	0.027	0.570 (0.346–0.939)

Note: 95% CI: 95% confidence intervals.

### Different expression of GNAL in different clinicopathologic characteristics

**Figure 3:**
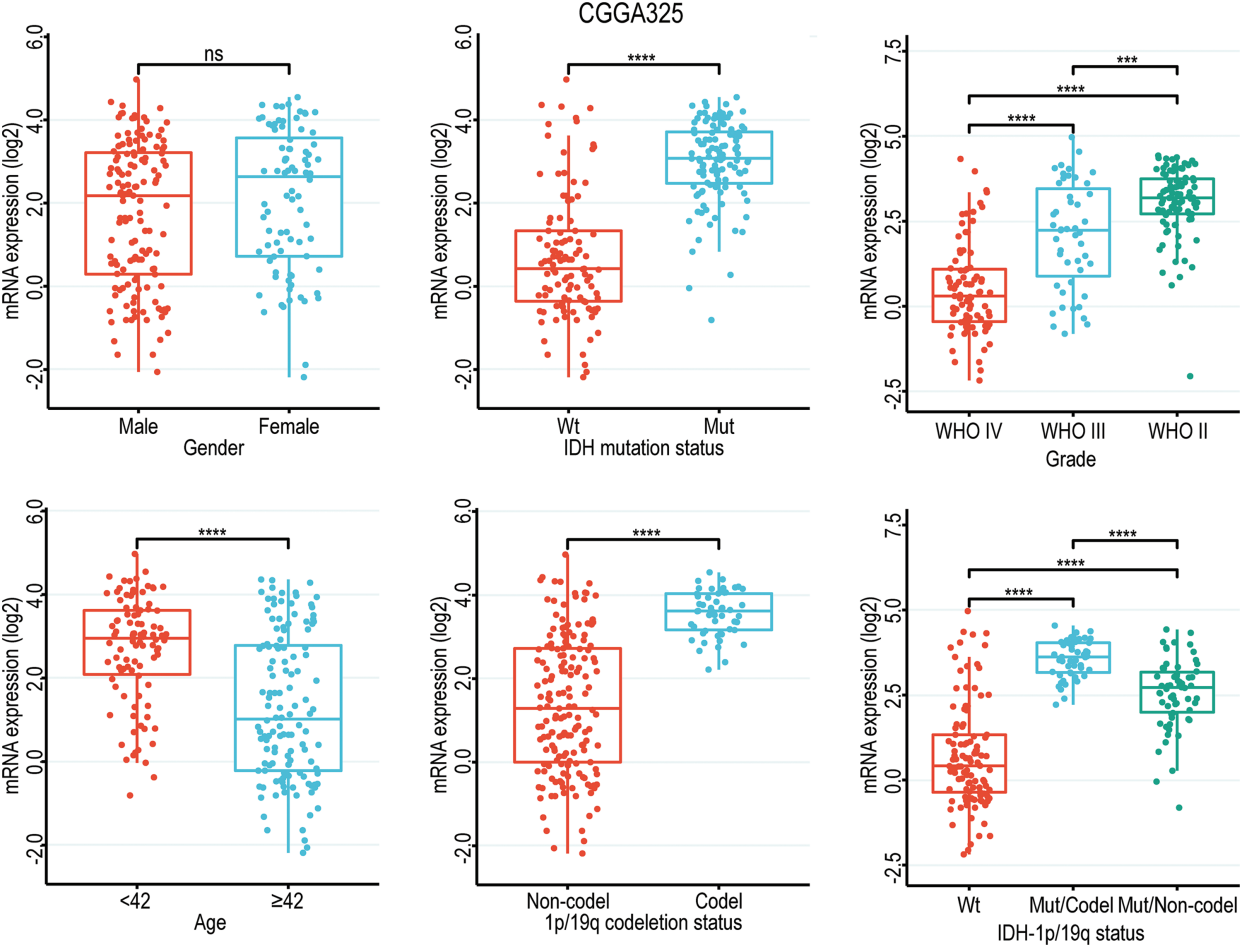
Box diagram of the relationship between *GNAL* and clinicopathological characteristics (including gender, age, grade, *IDH* mutation status, 1p/19q codeletion status, and *IDH* mutation status combined with 1p/19q codeletion status (*IDH*-1p/19q status)) in CGGA325 cohort. Wt, wildtype; Mut, mutation; Mut/Codel, *IDH* mutation combined with 1p/19q codeletion; Mut/Non-codel, *IDH* mutation combined with 1p/19q non-codeletion. The *p-*value is indicated in the figure. ns, no significance; ***p < 0.001; ****p < 0.0001.

**Figure 4:**
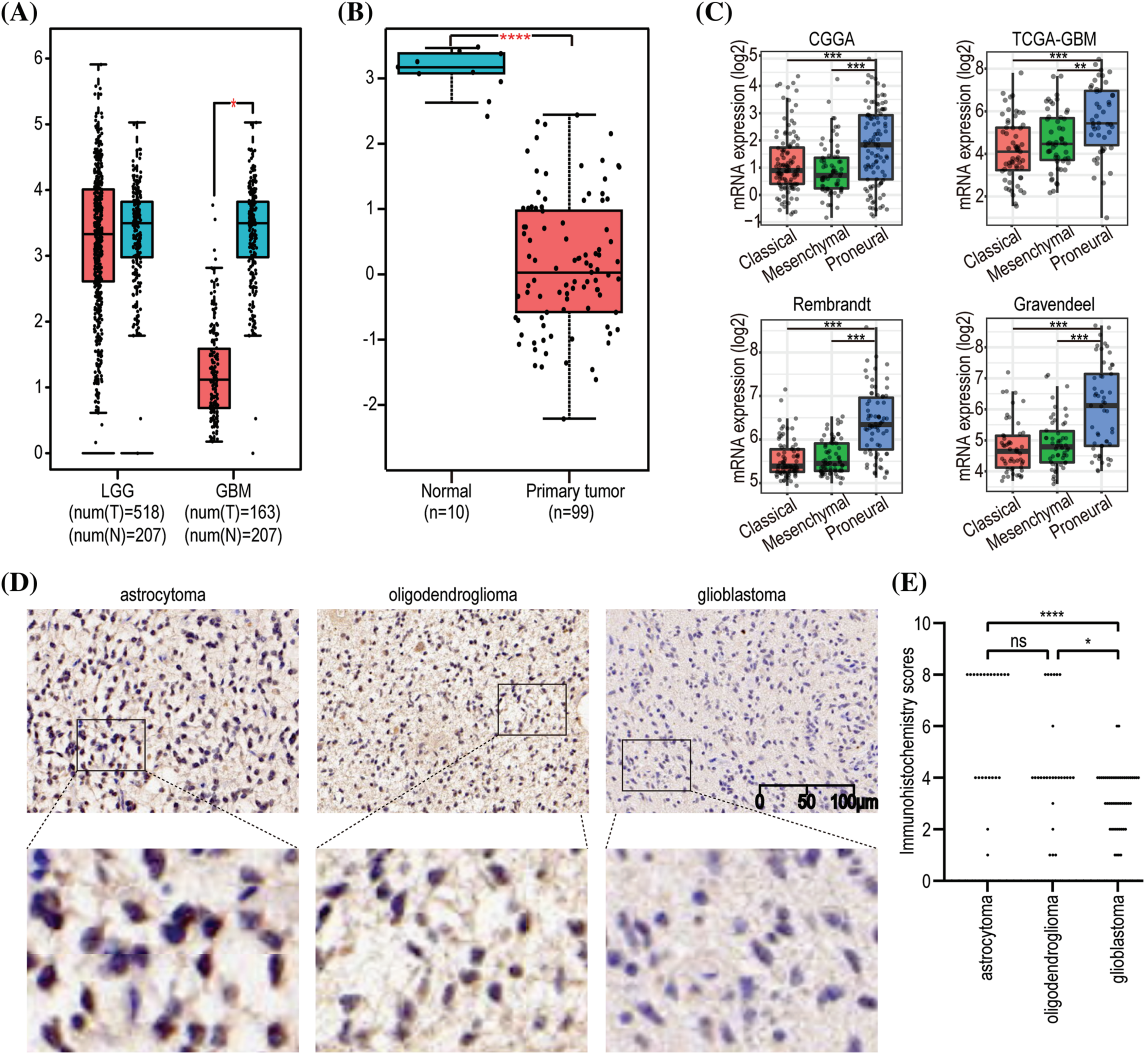
The expression pattern of *GNAL* in glioma. (A) Differences in *GNAL* mRNA expression in normal and cancerous tissues in LGG and GBM. (B) Differences in the expression of GNAL protein in normal and primary gliomas. (C) The expression differences in *GNAL* mRNA between different subtypes of GBM (including classical, mesenchymal, and procedural). (D) Representative immunohistochemistry (IHC) images for GNAL expression in astrocytoma, oligodendroglioma, and GBM. The scale bar is 100 µm. (E) The Kruskal-Wallis test was used to determine if the IHC staining score of one group in in astrocytoma (n = 23), oligodendroglioma (n = 27), and GBM (n = 51) had different distributions from the others, respectively. LGG: low-grade gliomas; GBM: glioblastoma. The *p-*value is indicated in the figure. ns, no significance; *p < 0.05; **p < 0.01; ***p < 0.001; ****p < 0.0001.

Given the close correlation between *GNAL* with clinicopathological characteristics and survival prognosis, we analyzed the differences in its expression under different clinicopathological characteristics. The *GNAL* expression were significantly higher in WHO grade II and grade III than grade IV (IV *vs*. III: *p* < 0.0001; IV *vs*. II: *p* < 0.0001; III *vs*. II: *p* < 0.001), young group (<42 *vs*. ≥42: *p* < 0.0001), *IDH* mutation than wildtype (Mut *vs*. Wt: *p* < 0.0001), 1p/19q codeletion than non-codeletion (Codel *vs*. Non-codel: *p* < 0.0001) in the CGGA325 cohort (Fig. 3). Similar analysis results were obtained in CGGA693 and TCGA-GBMLGG cohorts (Suppl. Fig. 2). To explore the differences of *GNAL* expression between normal tissue and glioma, analysis in GEPIA showed that *GNAL* expression is lower in GBM while there is no difference in LGG and normal tissue (Fig. 4A). The UALCAN database was used to further analyze the differential expression of GNAL protein in primary GBM and normal tissues. The results showed that GNAL protein was decreased in GBM (Fig. 4B). Altogether, the analysis of datasets in GlioVis showed that the expression of GNAL in the proneural group was higher than that in the mesenchymal group and the classical group (Fig. 4C). Furthermore, the immunohistochemical results showed that the staining score of GNAL protein in patients of astrocytoma and oligodendroglioma are stronger than GBM (Figs. 4D and 4E).

### Alterations in GNAL expression were associated with glioma development and progression

**Figure 5:**
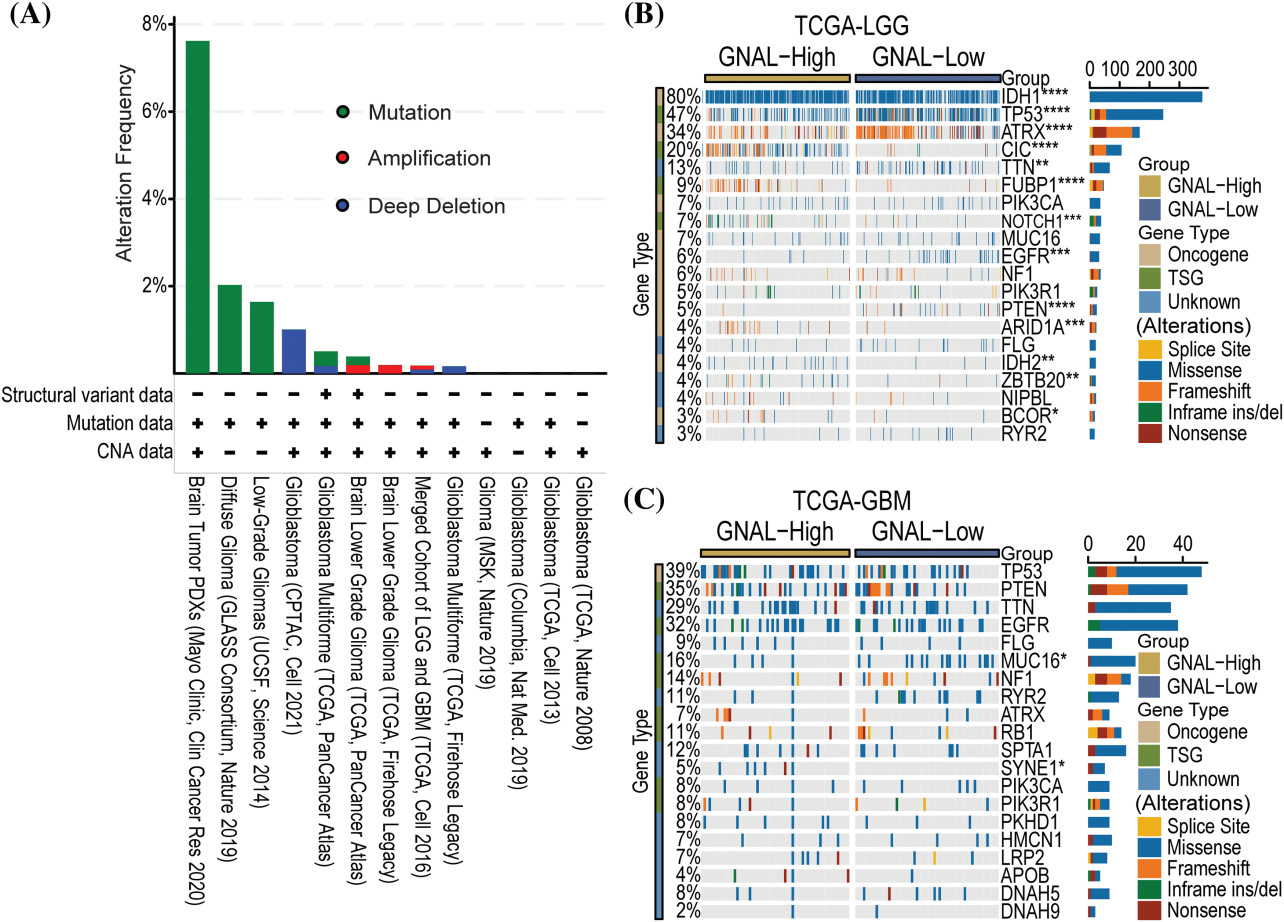
The relationship between *GNAL* and genetic alteration. (A) The alteration frequency with mutation type is displayed using the cBioPortal tool in glioma. Oncoplots of somatic mutant landscape in high and low *GNAL* expression groups in TCGA-LGG (B) and TCGA-GBM (C). The *p-*value is indicated in the figure. *p < 0.05; **p < 0.01; ***p < 0.001; ****p < 0.0001.

Gene alterations, such as mutations, deletions, or amplifications of oncogenes or tumor suppressor genes, are associated with tumor growth and progression [35]. Therefore, we analyzed the types of *GNAL* gene alterations, including mutations, amplifications, and deep deletions, in different glioma study cohorts using cBioPortal. The common alteration of *GNAL* in Brain Tumor PDXs (Mayo Clinic, Clin Cancer Res 2020) cohort was mutation (>6%). There was a 1% *GNAL* alteration with a deep deletion in Glioblastoma (CPTAC, Cell 2021) (Fig. 5A). We analyzed gene alterations based on Brain Tumor PDXs (Mayo Clinic, Clin Cancer Res 2020) cohort. The results showed that the mutant genes with significant significance between the wild-type and mutation *GNAL* groups included *FOXE1*, *DCP1B*, *MEOX2*, *SPRR3*, and *USF3* et al. (Suppl. Fig. 3). Then, the relationship between *GNAL* expression and specific genomic characteristics such as somatic mutations and copy number variations (CNVs) in TCGA-LGG and TCGA-GBM were analyzed using the Camoip database. Somatic mutation frequencies of *IDH1* (80%), *TP53* (47%), *ATRX* (34%), and *CIC* (20%) genes were elevated, and there were significant differences in somatic mutations of *IDH1*, *TP53*, *ATRX*, *CIC*, *TTN*, *FUBP1*, *NOTCH1*, *EGFR*, *PTEN*, *ARID1A*, *IDH2*, *ZBTB20*, and *BCOR* between the high and low expression groups of *GNAL* in TCGA-LGG (Fig. 5B). Somatic mutation frequencies for *TP53* (39%), *PTEN* (35%), *TTN* (29%), and *EGFR* (32%) were higher in TCGA-GBM. Significant difference in somatic mutations of *MUC16* and *SYNE1* was observed between the *GNAL*-high and low groups in the TCGA-GBM cohort (Fig. 5C). These results suggest a degree of genetic alterations of *GNAL* in glioma. Gene alterations in several oncogenes and suppressor genes were revealed in different groups of *GNAL* expression, suggesting that *GNAL* may be involved in glioma occurrence and development.

### GNAL regulates immune signaling in glioma

**Figure 6:**
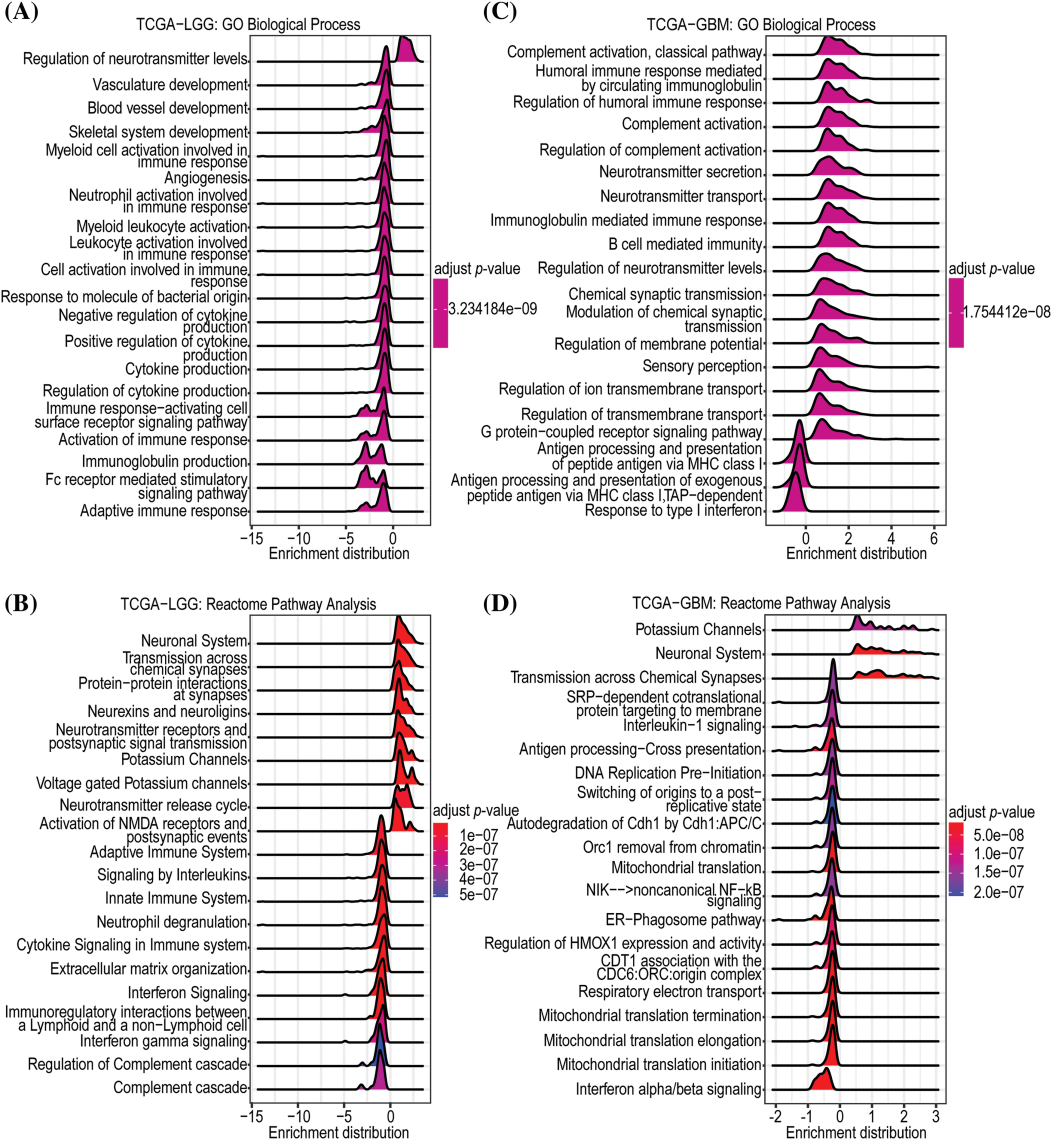
Enrichment analysis of *GNAL*. The GO-BP (A) and Reactome (B) pathway enrichment analyses in TCGA-LGG. And the GO-BP (C) and Reactome (D) pathway enrichment analyses in TCGA-GBM.

To establish the potential biological functions of *GNAL* in gliomas, GSEA analysis on Camoip was performed using TCGA-LGG and TCGA-GBM datasets. In LGGs, the Top 5 enriched GO-BPs included regulation of neurotransmitter levels, vasculature development, blood vessel development, skeletal system development, and myeloid cell activation-involved in immune responses (Fig. 6A). The Reactome pathways were mainly related to neuronal system, transmission across chemical synapses, protein-protein interactions at synapses, neurexins and neuroligins, neurotransmitter receptors and postsynaptic signal transmissions (Fig. 6B). The enriched GO-CCs were synaptic vesicle membrane, postsynaptic density, asymmetric synapse, synaptic vesicle, and cation channel complex (Suppl. Fig. 4A). The enriched GO-MFs were mainly involved in voltage-gated cation channel activity, voltage-gated channel activity, voltage-gated ion channel activity, gated channel activity, and potassium channel activity (Suppl. Fig. 4B). KEGG pathway enrichments were mainly related to nicotine addiction, glutamatergic synapse, synaptic vesicle cycle, GABAergic synapse, and morphine addiction (Suppl. Fig. 4C). In GBM, the enriched GO-BP (Top 5) included complement activation, classical pathway, humoral immune response mediated by circulating immunoglobulin, regulation of humoral immune response, complement activation, and regulation of complement activation (Fig. 6C). The Reactome pathway was mainly involved in potassium channels, neuronal system, transmission across chemical synapses, SRP-dependent cotranslational protein targeting to membrane, and interleukin-1 signaling (Fig. 6D). The enriched GO-CCs included immunoglobulin complex, immunoglobulin complex, circulating, synaptic vesicle membrane, axon terminus, and neuron projection terminus (Suppl. Fig. 4D). The enriched GO-MFs were mainly involved in immunoglobulin receptor binding, neurotransmitter receptor activity, voltage-gated cation channel activity, antigen binding, and voltage-gated potassium channel activity (Suppl. Fig. 4E). The enriched KEGG pathways were mainly involved in nicotine addiction, morphine addiction, GABAergic synapse, synaptic vesicle cycle, and glutamatergic synapse (Suppl. Fig. 4F). In conclusion, functional enrichment analysis revealed that *GNAL* may be involved in immunomodulatory responses in glioma.

### Relationship between GNAL expression and TIME

**Figure 7:**
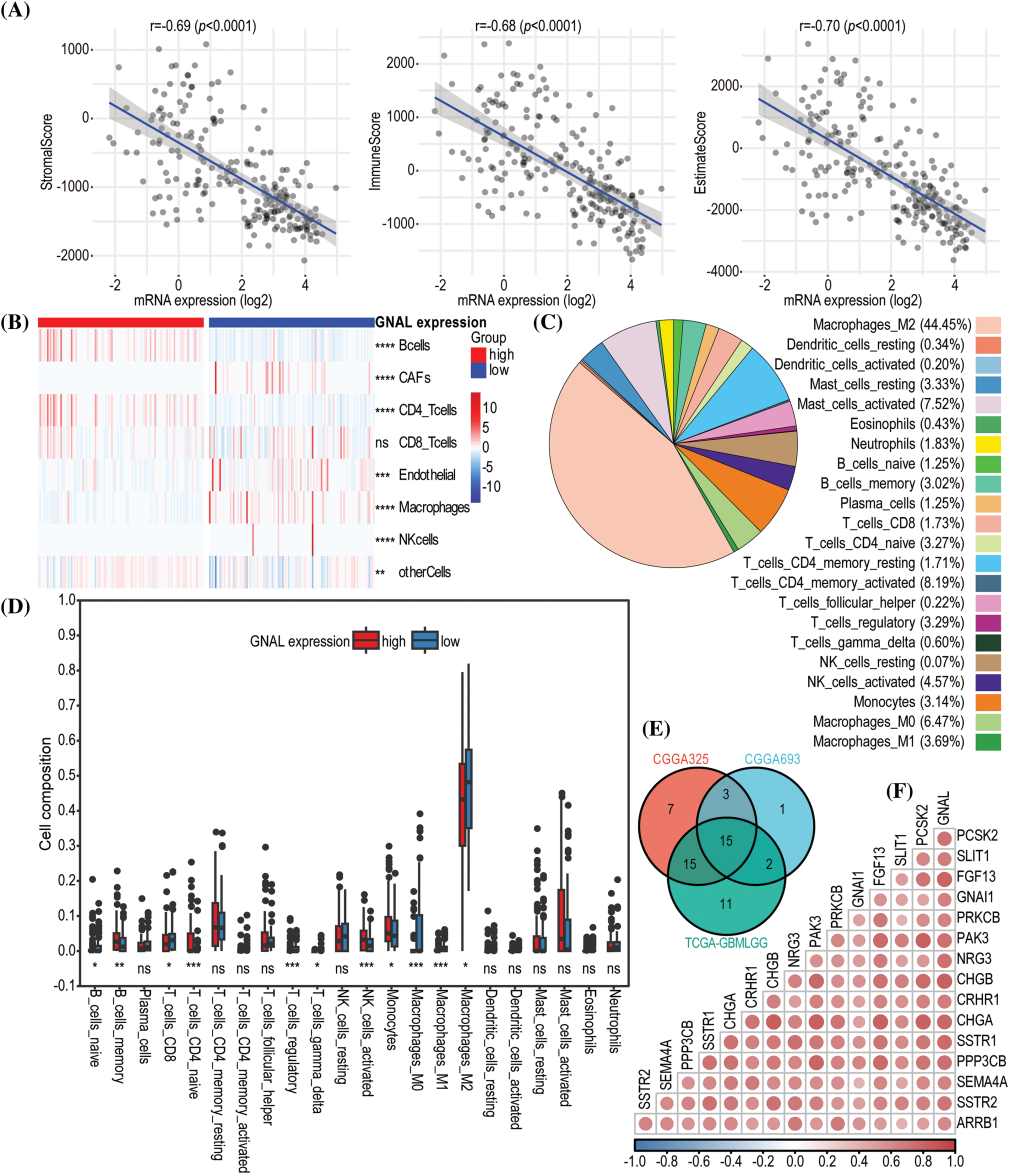
The immune infiltration analysis related to *GNAL* in glioma. (A) The correlations between *GNAL* mRNA levels and ESTIMATE scores (ImmueScore, StromalScore, and EstimateScore) in CGGA325 cohort. (B) A heatmap of correlations between *GNAL* mRNA levels and immune cells from CGGA325 cohort using the EPIC algorithm. (C) The proportion of 22 types of tumor-infiltrating immune cells from CGGA325 cohort using the CIBERSORT algorithm. (D) Comparison of immune cells infiltration between *GNAL* low and high expression groups in CGGA325 cohort. (E) A Venn diagram of screening highly immune-related genes. (F) A correlation heatmap of correlations between *GNAL* and highly immune-related genes from CGGA325 cohort. The *p-*value is indicated in the figure. ns: no significance; *p < 0.05; **p < 0.01; ***p < 0.001; ****p < 0.0001.

The above analyses suggest that *GNAL* may play an important role in tumor immunity. To assess the potential role of *GNAL* in TIME, we analyzed the effects of *GNAL* on tumor immunity using the immune infiltration score calculation tool in Sangerbox. In the CGGA325 cohort, the ESTIMATE algorithm revealed that low *GNAL* expression was accompanied by significantly higher StromalScore, ImmuneScore, and EstimateScore. With increasing *GNAL* expression, there were significant reductions in StromalScore, ImmuneScore, and EstimateScore, implying significant negative correlations (StromalScore: *r* = −0.69, *p* < 0.0001; ImmuneScore: *r* = −0.68, *p* < 0.0001 and EstimateScore: *r* = −0.70, *p* < 0.0001; Fig. 7A). These findings suggest that gliomas with low *GNAL* expression have higher immune and stromal cell infiltrations. Therefore, we investigated the relationship between *GNAL* expressions and tumor-infiltrating immune cells. Further analyses using the EPIC algorithm showed that infiltrations of B cells, cancer-associated fibroblasts (CAFs), CD4+ T cells, endothelial, macrophages, NK cells, and other cells were markedly different between *GNAL*-high and *GNAL*-low expression groups (Fig. 7B). The relationships between *GNAL* expression and 22 types of immune cells were analyzed using the CIBERSORT algorithm. Assessment of overall distributions of 22 types of immune cells revealed that M2 macrophages accounted for the highest proportions (44.45%) (Fig. 7C). The abundance of naive B cells, CD8 T cells, regulatory T cells (Tregs), gamma delta T cells, M0 macrophages, M1 macrophages, and M2 macrophages were significantly higher in the low *GNAL* expression group, relative to the high *GNAL* expression group. The abundance of memory B cells, naive CD4 T cells, activated NK cells, and monocytes in the low *GNAL* expression groups were lower than those in the high expression group (*p* < 0.05, Fig. 7D). In CGGA693 and TCGA-GBMLGG cohorts, *GNAL* levels were negatively correlated with StromalScore, ImmuneScore, and EstimateScore (Suppl. Figs. 5A and 5B). Heatmap of the EPIC algorithm result clarified the differences in tumor-infiltrating immune cells between the high and low *GNAL* expression groups (Suppl. Fig. 5C). The proportion of 22 types of tumor-infiltrating immune cells from CGGA693 and TCGA-GBMLGG cohorts is shown in Suppl. Fig. 5D. Intriguingly, the CIBERSORT algorithm revealed that some immune cells whose abundances were comparable in the CGGA325 cohort exhibited significant differences in CGGA693 or TCGA-GBMLGG (Suppl. Fig. 6A). These variations may be ascribed to the different sample sizes of glioma samples included in the dataset. Moreover, analysis of the TIMER database showed that in LGG, *GNAL* expression was negatively correlated with the infiltration of CD4+ cells (*r* = −0.433), macrophage (*r* = −0.394), neutrophil (*r* = −0.311), and dendritic cell (*r* = −0.348), respectively (Suppl. Fig. 6B).

To explore the relationship between *GNAL* and immune-related genes, 2483 immune-related genes were obtained from the ImmPortPortal database. The Veen map was used to reserve the genes that were common between the public cohorts, which were 1057 genes (Suppl. Fig. 6C). Fifteen immune genes that were highly positively correlated with *GNAL* were finally screened (Fig. 7E). Furthermore, a correlation heatmap was then constructed to indicate correlations between the 15 immune-related genes and *GNAL* (Fig. 7F). Correlation heatmaps revealed correlations between *GNAL* in CGGA693/ TCGA-GBMLGG and 15 immune-related genes (Suppl. Fig. 7). The correlation coefficients and *P* values are shown in Suppl. Table S6.

Tumor-infiltrating lymphocytes (TILs) are pivotal predictors of sentinel lymph node status and cancer survival [36]. To explore the correlation between TILs and *GNAL* in glioma, correlation heatmaps were downloaded from the TISIDB, and immunoinhibitors, immunostimulators, MHC molecules, chemokines receptors, chemokines were also observed (Suppl. Fig. 8). Interestingly, *GNAL* levels were negatively correlated with most TILs and MHC molecules in LGG. The same trend was observed between *GNAL* levels and several immunoinhibitors (*HAVCR2*, *IL10RB*, *LGALS9*, *PDCD1LG2*, and *TGFB1*), immunostimulators (*CD276*, *CD40*, *CD86*, *MICB*, and *TNFRSF14*), chemokines receptors (*CCR1*, *CCR5*, *CXCR2*, *CXCR4*, and *CXCR6*) and chemokines (*CCL2*, *CCL5*, *CCL22*, *CXCL10*, and *CXCL16*).

### More Sensitivity to immunotherapy for GNAL low-expression group and GNAL can effectively enhance the inhibitory effect of anti-tumor drugs

**Figure 8:**
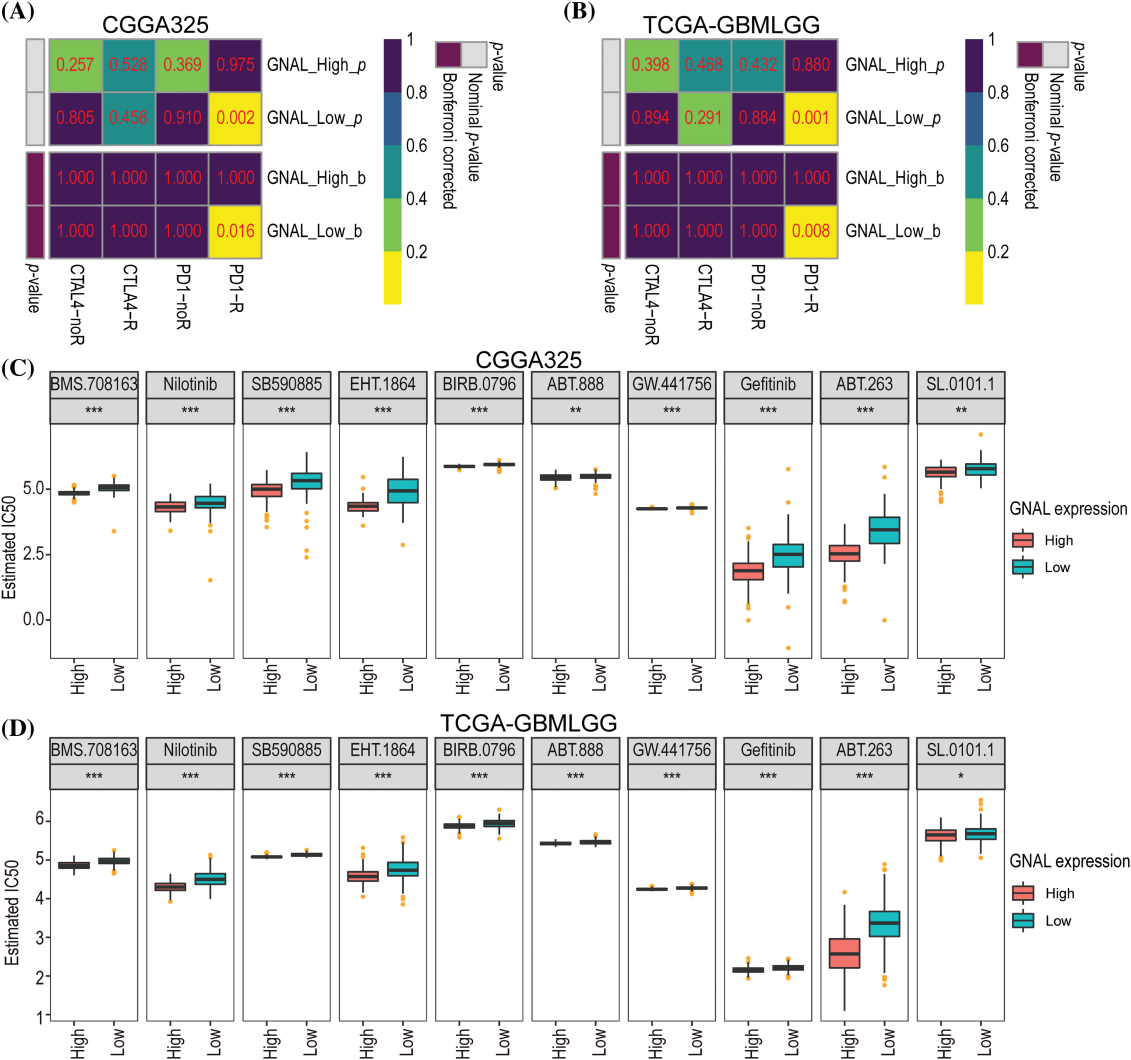
Differential putative chemotherapeutic and immunotherapeutic response. Submap analysis manifested that *GNAL* low expression group could be more sensitive to the anti-PD-1 therapy in CGGG325 cohort (A) and TCGA-GBMLGG cohort (B). The box plots of the estimated IC50 for 10 selected drugs are shown in CGGG325 cohort (C) and TCGA-GBMLGG cohort (D) for *GNAL* high and low-expression groups. IC50: half-maximal inhibitory concentration. The *p-*value is indicated in the figure. *p < 0.05; **p < 0.01; ***p < 0.001.

To determine whether *GNAL* can be a predictive biomarker of chemotherapy or immunotherapy response, we attempted to assess the correlation between different expression groups of *GNAL* and responsiveness. The results demonstrated that the *GNAL* low-expression group exhibited efficacy with anti-PD-1 therapy (Bonferroni-corrected *p* = 0.016) in the CGGG325 cohort and validated it in the TCGA-GMBLGG cohort (Bonferroni-corrected *p* = 0.008) (Figs. 8A and 8B). The CGGA693 cohort (Suppl. Fig. 9A) also showed consistent results. To further compare the response of the *GNAL* high-expression group and low-expression group to potential drugs, the predictive model was trained on the GDSC cell line data set using ridge regression, yielding a satisfactory predictive accuracy as evaluated by 10-fold cross-validation. A total of 139 potential drugs were screened, and the IC50 of each sample in the above cohorts was estimated based on the predictive models of these drugs. Finally, 10 drugs (BMS.708163: γ-secretase inhibitor; Nilotinib: Bcr-Abl inhibitor; SB590885: B-Raf/c-Raf inhibitor; EHT.1864: Rac family GTPase inhibitor; BIRB.0796: p38 MAPK inhibitor; ABT.888: PARP-1/2 inhibitor; GW.441756: TrkA inhibitor; Gefitinib: EGFR inhibitor; ABT.263: Bcl-xL/2/w inhibitor and SL.0101.1: tRSK inhibitor) which showed inter-group differences in all cohorts were selected for display (Figs. 8C and 8D, Suppl. Fig. 9B). A significant difference in the estimated IC50 between the *GNAL* high and low-expression groups for these chemotherapy drugs was observed. Among them, the high expression group of *GNAL* exhibited a lower estimated IC50, indicating that *GNAL* can effectively enhance the inhibitory effect of anti-tumor drugs.

### DNA methylation levels of GNAL in glioma

**Figure 9:**
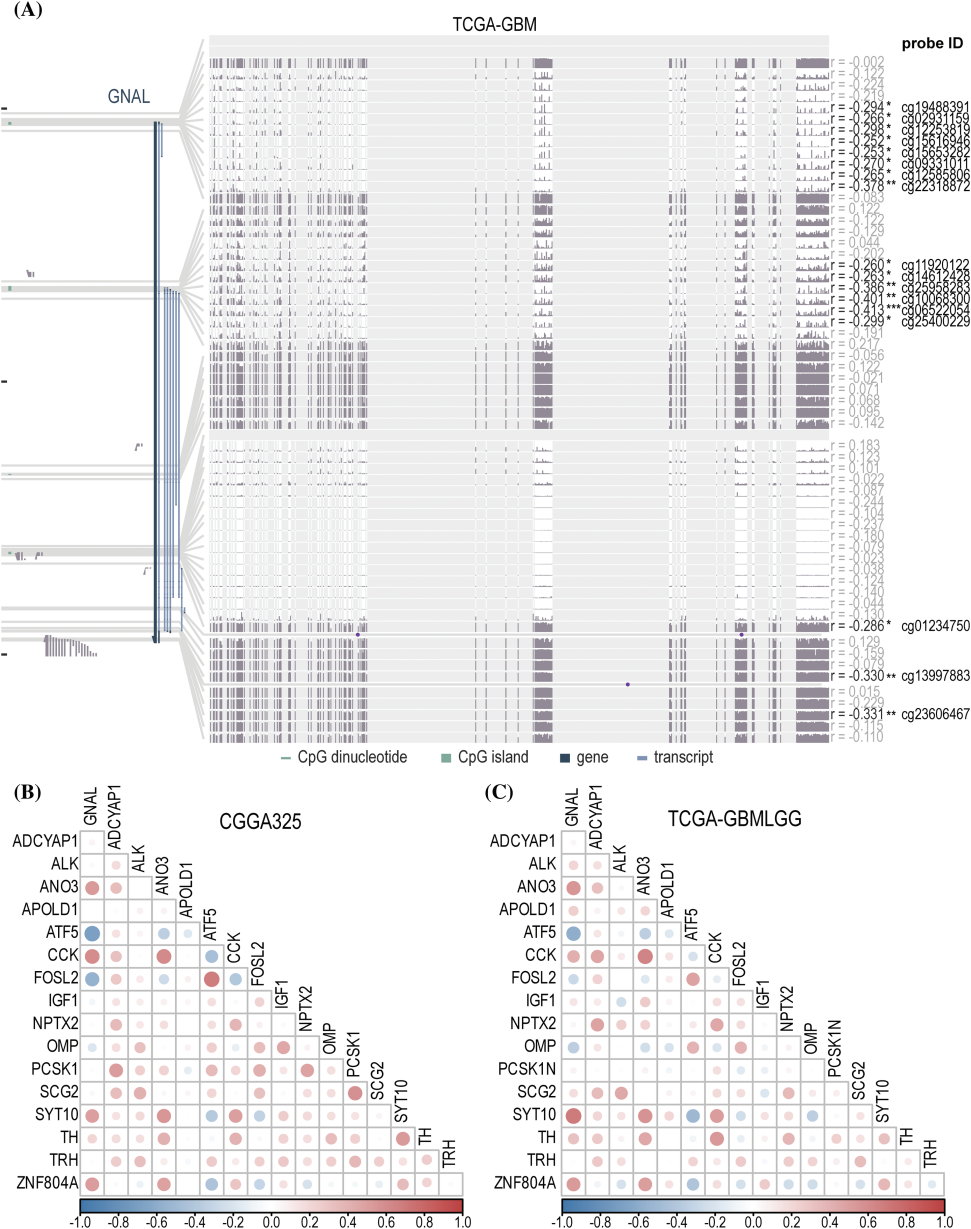
The analysis of DNA methylation and related naris-occlusion controlled genes (NOCGs). (A) *GNAL* expression was negatively correlated with *GNAL* DNA methylation in TCGA-GBM across MEXPRESS. The correlation heatmap of correlations between *GNAL* and NOCGs from CGGA325 cohort (B) and TCGA-GBMLGG cohort (C). The *p-*value is indicated in the figure. *p < 0.05; **p < 0.01; ***p < 0.001.

DNA hypomethylation of oncogenes increases their expression thereby promoting tumor development [37]. To explore the DNA methylation levels of *GNAL* in glioma, the MEXPRESS database analysis revealed a negative correlation between mRNA expression of *GNAL* in GBM and its methylation levels (Fig. 9A). Probe ID: cg06522054 (*r* = −0.413, *p* < 0.001) revealed negative correlations. Suppl. Fig. 10A shows that in LGG, *GNAL* mRNA expression at most probes was negatively correlated with its methylation levels, while correlations were positive at some probes (for example, cg0341788: *r* = 0.461, *p* < 0.001). In summary, elevated methylation levels of *GNAL* may contribute to the suppression of its expression in glioma.

### Correlations between GNAL and NOCGs

Gliomas originating in oligodendrocyte precursor cells (OPCs) preferentially appear in OB, and glioma development is subsequently affected by manipulation of the activities of olfactory receptor neurons (ORNs), which then spread to another brain parenchyma [13]. Chen et al. deprived the normal olfactory experience of termed conditional knockout (CKO) mice using the naris occlusion method and found 17 genes that were consistently downregulated in OB. To establish the relationship between 17 NOCGs and *GNAL* in glioma, we assessed its correlations with *GNAL* in CGGA325 (Fig. 9B) and TCGA-GBMLGG (Fig. 9C). Correlation heatmap for CGGA693 was shown in Suppl. Fig. 10B. In summary, the results demonstrated that the *GNAL* gene was negatively correlated with *ATF5* and *FOSL2*, and positively correlated with *CCK* and *ZNF804A*.

## Discussion

*GNAL*, one of the hub-gene related to prognosis in glioma [19]. However, its potential as an independent prognostic indicator, the expression of its protein level in glioma pathological tissues, and its correlation with the tumor immune microenvironment and treatment response remain unknown. By determining the prognostic values of *GNAL* in glioma and its association with clinicopathologic characteristics, we found that glioma patients with high *GNAL* expression had more significant outcomes. Meanwhile, the integration of routinely employed clinicopathological parameters demonstrates a robust predictive value in predicting prognosis [38], thereby furnishing a sound theoretical basis for personalized treatment strategies tailored to glioma patients. In this study, multivariate Cox regression analysis revealed that the *GNAL* may serve as independent prognostic predictor, whereas *IDH* status and 1p/19q status are not independent prognostic factors. Multivariate Cox regression analysis combining clinicopathological characteristics and *GNAL* expression revealed that *GNAL* is an independent prognostic indicator. Moreover, *GNAL* was found to be highly expressed in some glioma patients with favorable molecular biomarkers, such as *IDH* mutation and 1p/19q codeletion. These results suggest that higher *GNAL* expression is associated with improved prognostic outcomes and closely linked to clinicopathological characteristics. This implies that *GNAL* has the potential to be a prognostic biomarker for glioma.

Meanwhile, TIME may be a significant factor leading to poor prognosis in glioma patients [39]. The findings of several recent studies have indicated that *OLFML3*, *EVA1B*, and *FERMT3* are potential prognostic markers for glioma [40–42], which are significantly associated with poor prognosis and tumor immune microenvironment. In our research, we examined the entirety of immune infiltration and analyzed a specific subset of immune-infiltrated cells, ultimately discovering that *GNAL* was intimately linked to tumor immunity. Findings from the ESTIMATE algorithm revealed significant negative correlations between *GNAL* expression and StromalScore, ImmuneScore, and EstimateScore, implying that glioma patients with low *GNAL* expression may not mount strong anti-tumor immune responses that can then induce immune escape. We conducted a further investigation into the variations in immune cell infiltrations across different *GNAL* expression levels. The EPIC algorithm showed that CAFs and macrophages were enriched in the *GNAL* low-expression group. The CIBERSORT algorithm revealed that M2-type accounts for 44.45% of the 22 types of immune cells whereas M1-type macrophages only account for about 0.66% and M2-type are also enriched in the *GNAL* low-expression group in the CGGA325 cohort. Vidyarthi et al. identified M2-type as the dominant subtype of tumor-associated macrophages (TAMs) in the TIME of the high-grade glioma, suggesting that it may have important roles in immune escape, tumor recurrence, drug resistance, and malignant transformation in glioma [43]. Previous studies have demonstrated that patients with the high-CAFs subtype have a poorer prognosis compared to those with the low-CAFs subtype. Additionally, it has been observed that the group with a high CAFs-related gene risk score shows more favorable responses to anti-PD-1 treatment [44].

Glioma has been described as a “cold” tumor that does not respond well to immunotherapy based on patient’s response [45]. The *GNAL* low-expression group was found to have a more abundant immune cell population in the immune infiltration analysis, suggesting that the corresponding patients might exhibit increased sensitivity to immunotherapy. Therefore, we further used the submap algorithm to explore the sensitivity of different expression groups of *GNAL* to immune checkpoint blockers and found that patients with *GNAL* low-expression showed higher responses to anti-PD1 treatment. Subsequently, we identified 10 targeted drugs with significant differences between the *GNAL* high and low-expression groups. A preclinical study demonstrated nilotinib may be effective for the management of a platelet-derived growth factor receptor alpha (PDGFRα)-dependent group of pediatric gliomas [46]. While EHT.1864 was confirmed to reduce the infiltration propensity of GBM [47]. As for ABT.888 (veliparib) and gefitinib, it has been confirmed by a large number of research reports that they can help improve the treatment of glioma [48,49]. ABT.263 (navitoclax) as a Bcl-xL/Bcl-2/Bcl-w inhibitor, potentiated caspase-dependent cell death in response to 2-deoxyglucose or its combination with metformin in pediatric glioma [50]. In recent years, clinical trials have investigated the prognostic outcome of glioma patients receiving gefitinib [51], ABT-888 (veliparib) [52], and nilotinib (ClinicalTrials.gov ID: NCT01140568). Although there has been no substantial improvement in prognosis, this attention fosters the development of personalized targeted therapy for gliomas. These findings demonstrate that *GNAL* is a potential promising therapeutic target in glioma.

Furthermore, we found a strong positive correlation between *GNAL* and some immune-related genes. Among them, *SEMA4A* has been reported to be involved in the induction of apoptosis of human oligodendrocytes by regulating the immune system [53]. *FGF13* and *PRKCB* act as antioncogenes that inhibit tumor progression by modulating the immune functions in acute myeloid leukemia and lung adenocarcinoma [54,55]. Residual genes have not been identified as independent factors but rather as complementary genes that synergistically contribute to tumor development and progression. Therefore, further analysis is needed to explore the signaling pathway regulated by *GNAL*. In summary, immune infiltration analysis showed that *GNAL* was closely related to the development of an immunosuppressive microenvironment in glioma.

Studies have found that DNA methylation contributes to the occurrence and development of tumors through epigenetic regulation [56]. Kang et al. suggested that DNA methylation may improve the application of individualized therapy in GBM patients and influence the prognosis of patients as a predictor [57]. Therefore, we explored the relationship between *GNAL* expression and its DNA methylation level in glioma. A significant negative correlation was observed between all probes in LGGs and most probes in GBMs, suggesting that the low expression of *GNAL* in glioma may have been affected by its DNA methylation.

Nose-to-brain delivery is an attractive non-invasive pathway for glioma targeted therapy, which involves the transport of drugs to the brain through the nasal mucosa [12,58]. These medications can access the CNS through perivascular channels within the lamina propria or by employing intracellular and extracellular pathways involving the olfactory and trigeminal nerves [59,60]. As for olfaction, a newly discovered risk factor for accelerating the progression of glioma, detected 17 target genes that exhibited consistent downregulation after naris occlusion [16]; among them, *ATF5* and *FOSL2* were negatively correlated with *GNAL*. As an anti-apoptotic protein, *ATF5* is highly expressed in neuroblastoma, medulloblastoma, and glioblastoma, it promotes cancer cell survival [61,62]. Besides, *FOSL2* is closely associated with the malignant progression of GBM [63]. Further studies should explore whether *ATF5*/*FOSL2* and *GNAL* compete or inhibit each other upstream or downstream during the development of glioma.

Our current study demonstrates the potential of *GNAL* as an independent prognostic indicator. However, additional functional experimental verification is necessary to establish its role as a tumor suppressor in glioma. It is important to note that the assessment of immune cell abundance was conducted solely through the use of algorithm-based evaluations. Therefore, the experimental verification of the relationship between *GNAL* expression and immune cell expression, chemotherapy response, or immunotherapy response in gliomas is imperative. Although the analysis of pathway enrichment revealed that *GNAL* was associated with several immune-related pathways, there may be other pathways in which *GNAL* plays an important role, such as the closely related cAMP signaling pathway [64]. Studies have demonstrated that the cAMP, which acts as a second signaling molecule, can inhibit the progression of glioma [65], and decreased expression of *GNAL* may be the main reason for the dysregulation of this pathway in glioma.

## Supplementary Materials

Supplementary Fig. 1 The forest plots of univariate analysis from CGGA325 (**A**), CGGA693 (**B**), and TCGA-GBMLGG (**C**) cohorts. 95% CI: 95% confidence intervals.

Supplementary Fig. 2 The relationship between *GNAL* and clinicopathological characteristics (including gender, age, grade, *IDH* mutation status, 1p/19q codeletion status, and *IDH* mutation combined with 1p/19q codeletion status (*IDH*-1p/19q status)) in CGGA693 (**A**) and TCGA-GBMLGG (**B**) cohorts. Wt: wildtype; Mut: mutation; Mut/Codel: *IDH* mutation combined with 1p/19q codeletion; Mut/Non-codel: *IDH* mutation combined with 1p/19q non-codeletion. The p-value is indicated in the figure. ns: no significance; *: *p*<0.05; ***: *p*<0.001; ****: *p*<0.0001.

Supplementary Fig. 3 The somatic mutant landscape in wild-type and mutation *GNAL* groups based on Brain Tumor PDXs (Mayo Clinic, Clin Cancer Res 2020) cohort in CBioPortal. PDX: Patient-derived tumor xenograft. The p-value is indicated in the figure. *: *p*<0.05.

Supplementary Fig. 4 The GO-CC (**A**), GO-MF (**B**), and KEGG (**C**) pathway enrichment analyses in TCGA-LGG. The GO-CC (**D**), GO-MF (**E**), and KEGG (**F**) pathway enrichment analyses in TCGA-GBM. GO-CC: gene ontology-cellular component; GO-MF: gene ontology-molecular function; KEGG: kyoto encyclopedia of genes and genomes.

Supplementary Fig. 5 The correlations between *GNAL* and ImmueScore, StromalScore, and EstimateScore from CGGA693 (**A**) and TCGA-GBMLGG (**B**) cohorts. (**C**) A heatmap of correlations between *GNAL* and immune cells from CGGA693 and TCGA-GBMLGG cohorts using the EPIC algorithm. (**D**) The proportion of 22 types of tumor-infiltrating immune cells from CGGA693 and TCGA-GBMLGG cohorts using the CIBERSORT algorithm. The p-value is indicated in the figure. ns: no significance; ****: *p*<0.0001.

Supplementary Fig. 6 (**A**) The correlations between *GNAL* and 22 types of tumor-infiltrating immune cells from CGGA693 and TCGA-GBMLGG cohorts using the CIBERSORT algorithm. (**B**) Correlation between *GNAL* expression and immune cells in LGG and GBM from TIMER database. (**C**) A Venn diagram of screening immune-related genes. LGG: low-grade gliomas; GBM: glioblastoma. The p-value is indicated in the figure. ns: no significance; *: *p*<0.05; **: *p*<0.01; ***: *p*<0.001.

Supplementary Fig. 7 A correlation heatmap of correlations between *GNAL* and highly immune-related genes from CGGA693 and TCGA-GBMLGG cohorts.

Supplementary Fig. 8 The relationship between *GNAL* mRNA levels and abundance of immunoinhibitors (**A**), immunostimulators (**B**), MHC molecules (**C**), chemokine receptors (**D**), chemokines (**E**), and TILs (**F**) in LGG and GBM using TISIDB. MHC: major histocompatibility complex; TIL: Tumor-infiltrating lymphocytes; LGG: low-grade gliomas; GBM: glioblastoma.

Supplementary Fig. 9 Differential putative chemotherapeutic and immunotherapeutic response. Submap analysis manifested that *GNAL* low expression group could be more sensitive to the anti-PD-1 therapy in CGGG693 cohort (**A**). The box plots of the estimated IC50 for 10 selected drugs are shown in CGGG693 cohort (**B**) for *GNAL* high and low-expression groups. IC50: half-maximal inhibitory concentration. The p-value is indicated in the figure. *: p<0.05; ***: p<0.001.

Supplementary Fig. 10 (**A**) *GNAL* expression was associated with *GNAL* DNA methylation in TCGA-LGG across MEXPRESS. The correlation heatmaps of correlations between *GNAL* and NOCGs from CGGA693 cohort (**B**). NOCG: naris-occlusion controlled genes. The p-value is indicated in the figure. *: p<0.05; **: p<0.01; ***: p<0.001.

## Data Availability

The data supporting reported results can be found in CGGA (http://www.cgga.org.cn/index.jsp).

## Supplementary Materials

The supplementary material is available online at https://doi.org/10.32604/or.2023.045769.
