# Supplementary figures and images for: Multi-cohort comprehensive analysis unveiling the clinical value and therapeutic effect of GNAL in glioma
Source: Oncol Res. 2024 Apr 23;32(5):965–81. doi: 10.32604/or.2024.045769 (PMC11055992; doi:10.32604/or.2024.045769)

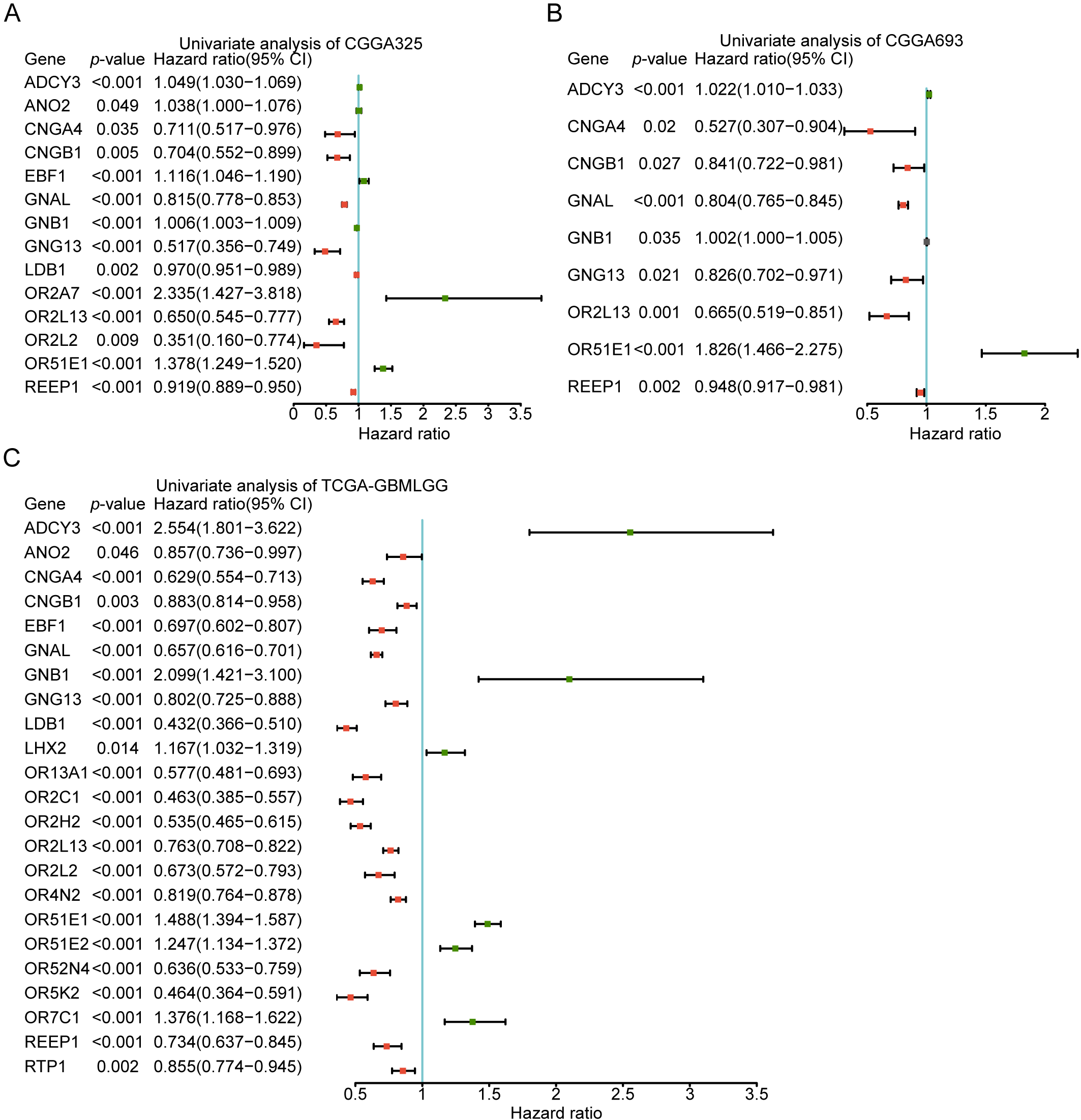

Supplement: Supplementary Fig. 1 [file OncolRes-32-45769-s001.tif]

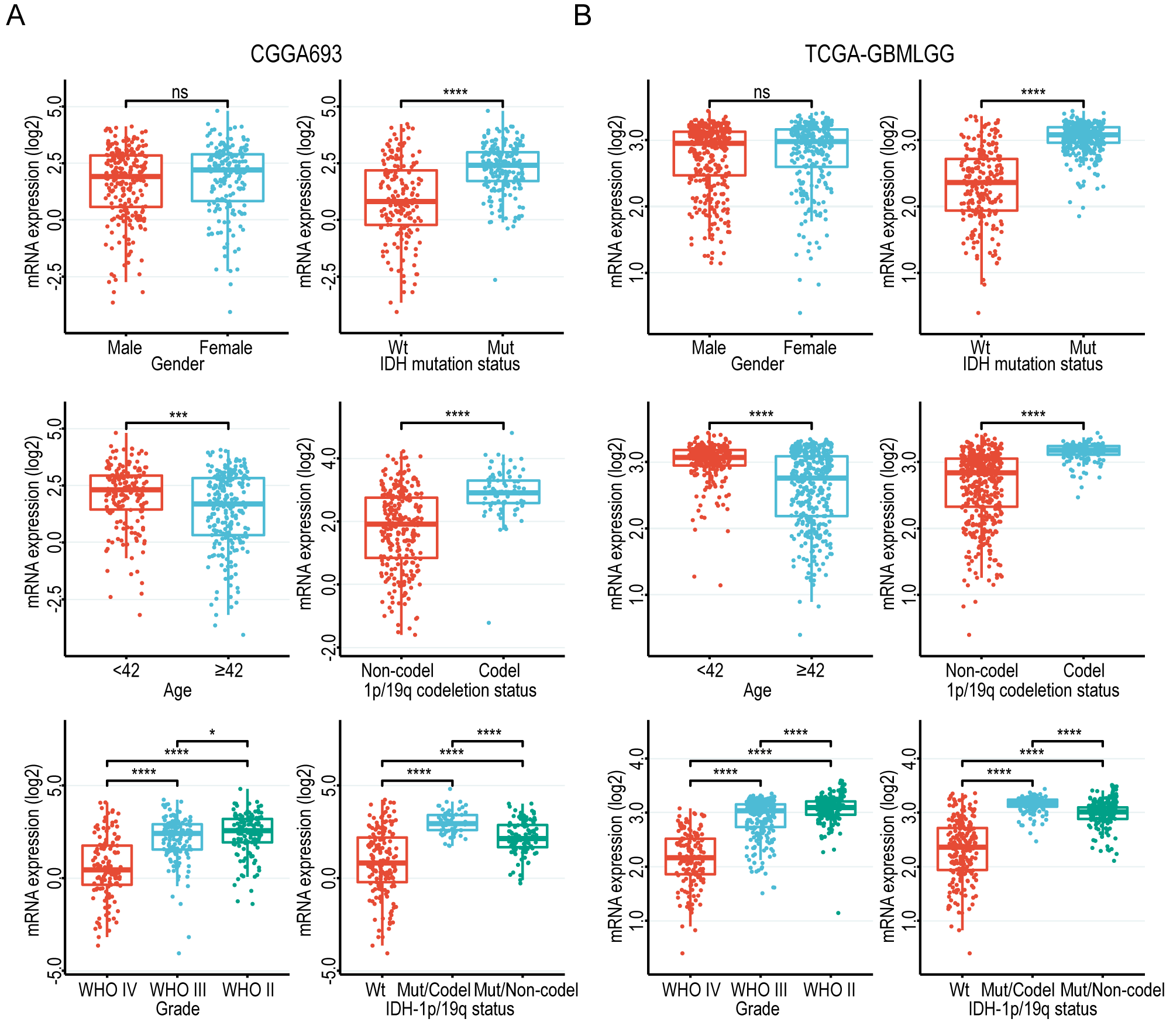

Supplement: Supplementary Fig. 2 [file OncolRes-32-45769-s002.tif]

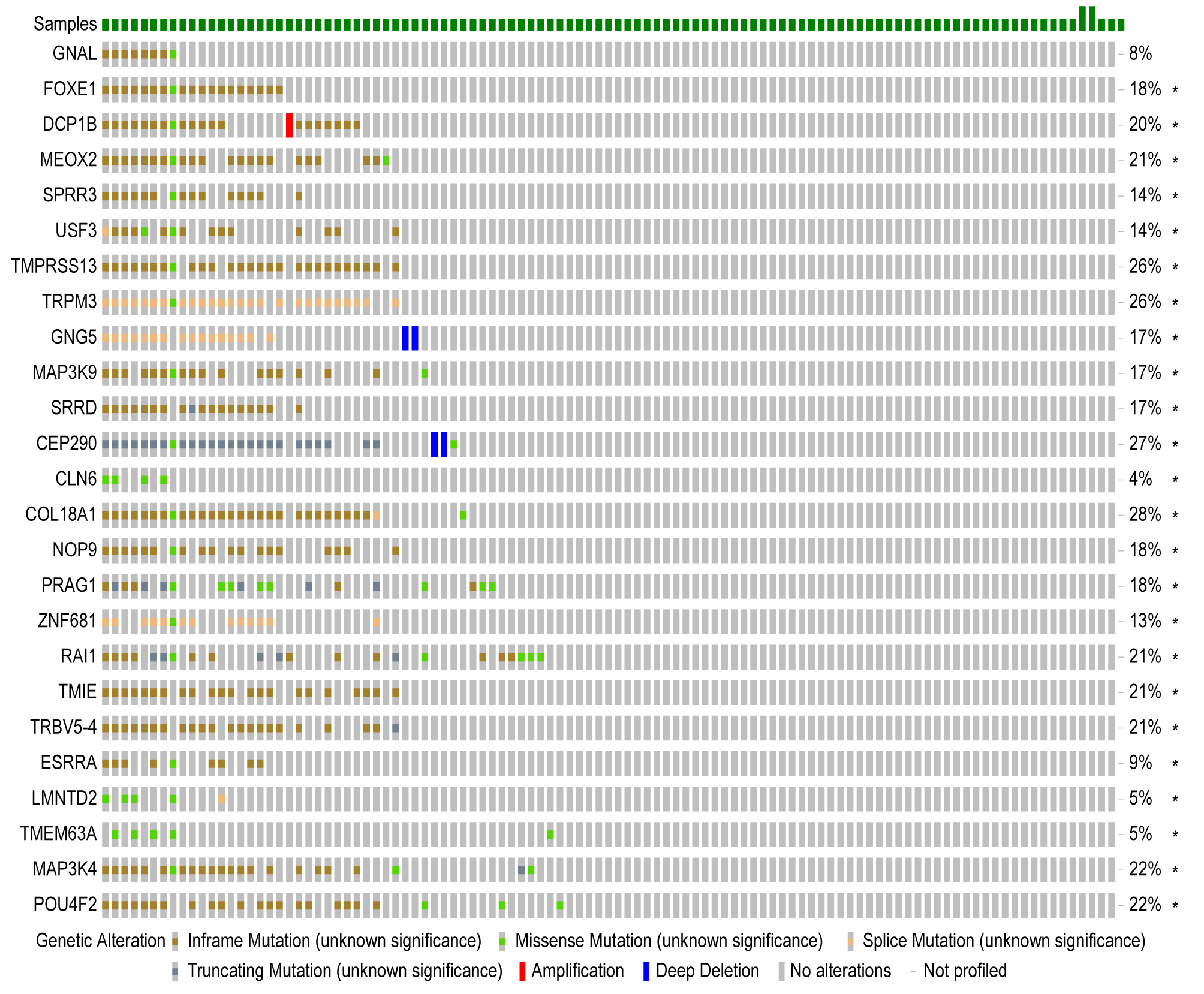

Supplement: Supplementary Fig. 3 [file OncolRes-32-45769-s003.tif]

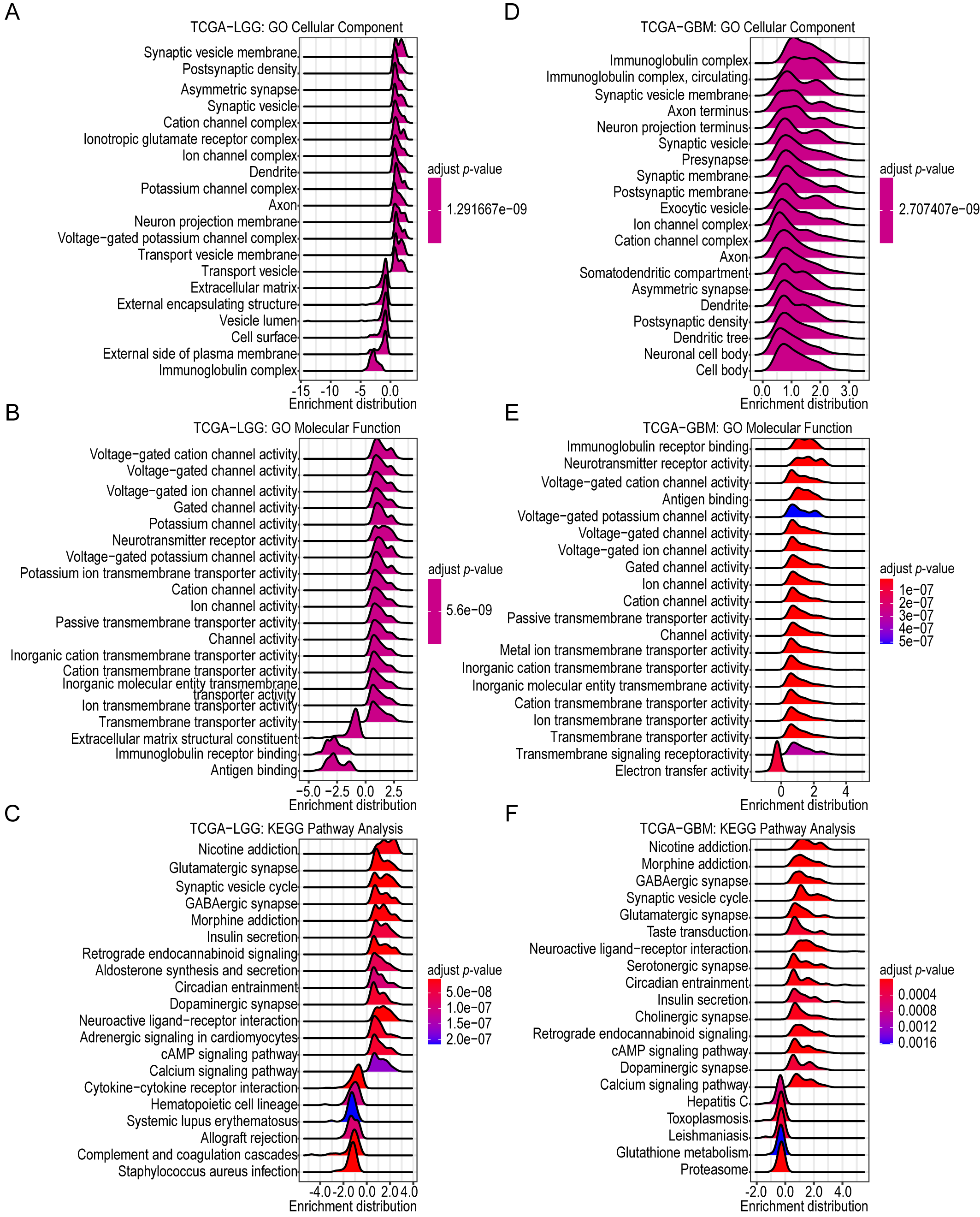

Supplement: Supplementary Fig. 4 [file OncolRes-32-45769-s004.tif]

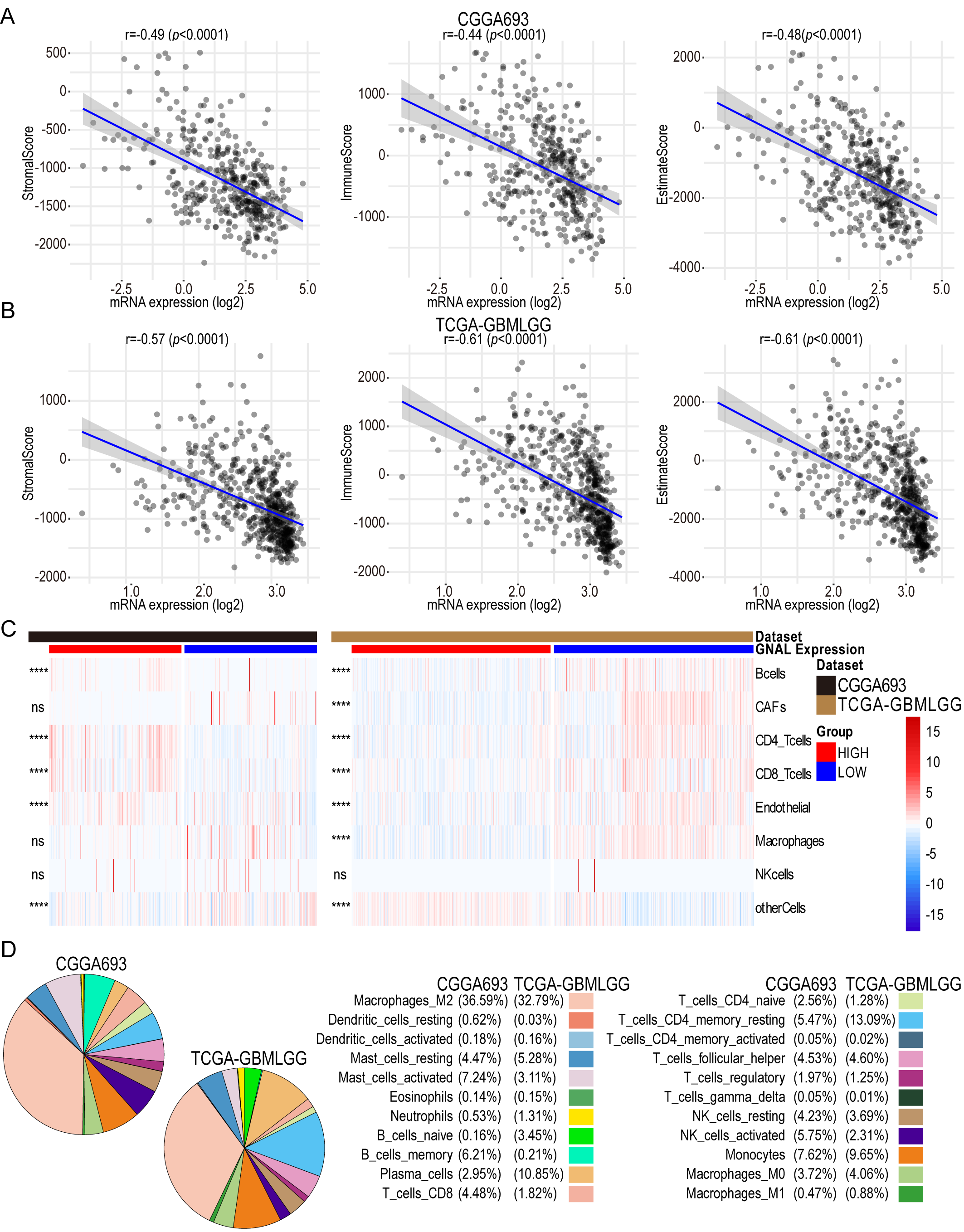

Supplement: Supplementary Fig. 5 [file OncolRes-32-45769-s005.tif]

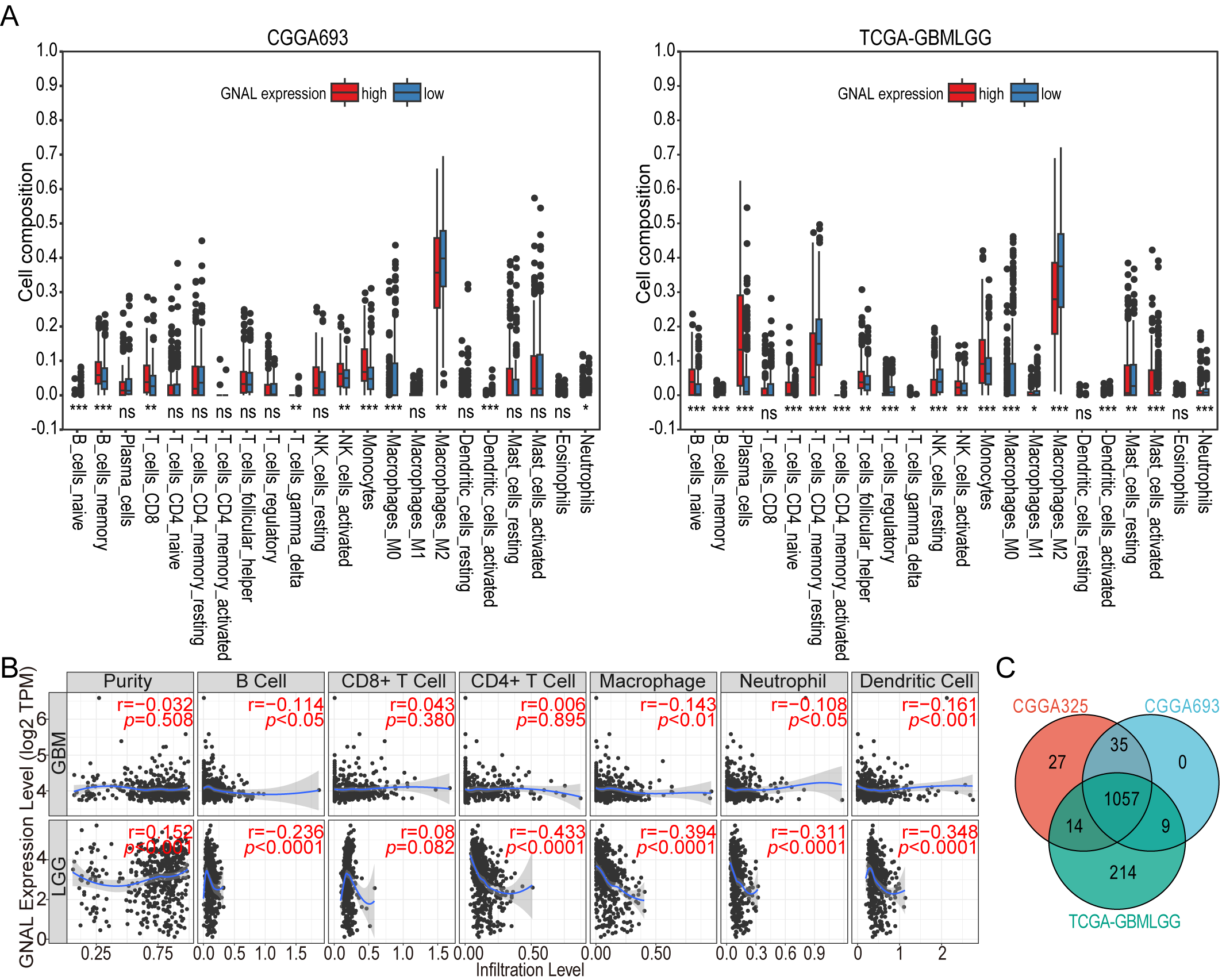

Supplement: Supplementary Fig. 6 [file OncolRes-32-45769-s006.tif]

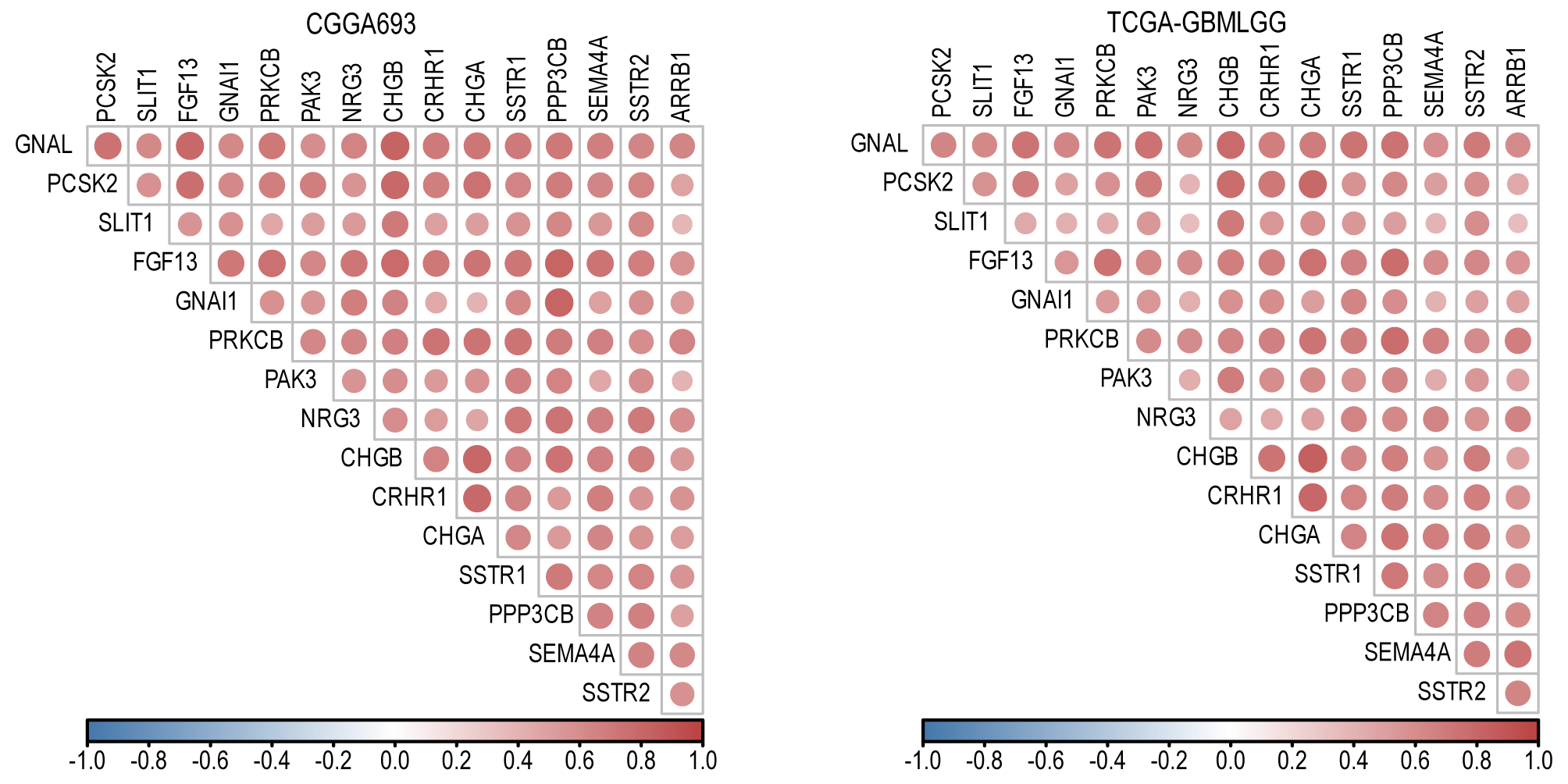

Supplement: Supplementary Fig. 7 [file OncolRes-32-45769-s007.tif]

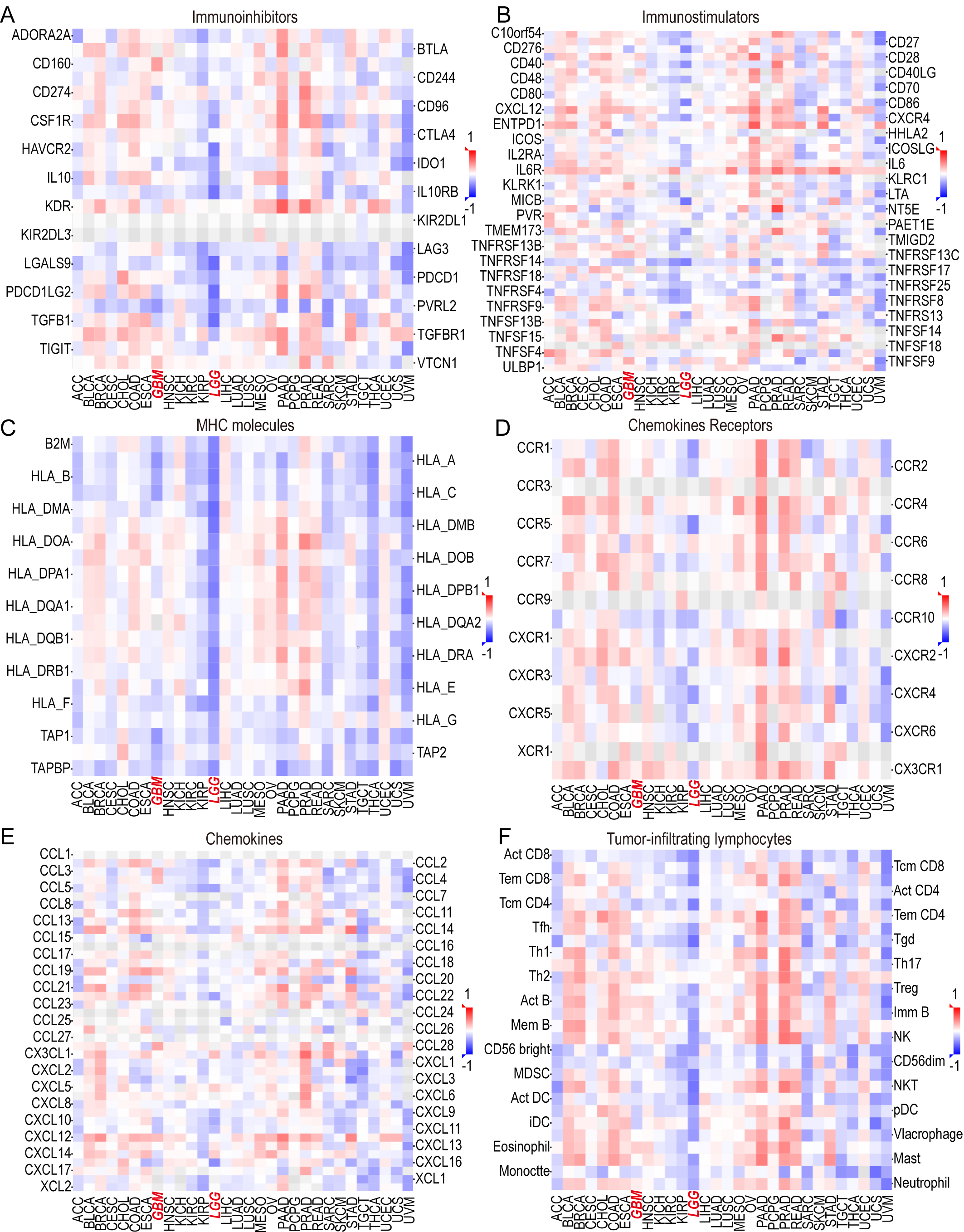

Supplement: Supplementary Fig. 8 [file OncolRes-32-45769-s008.tif]

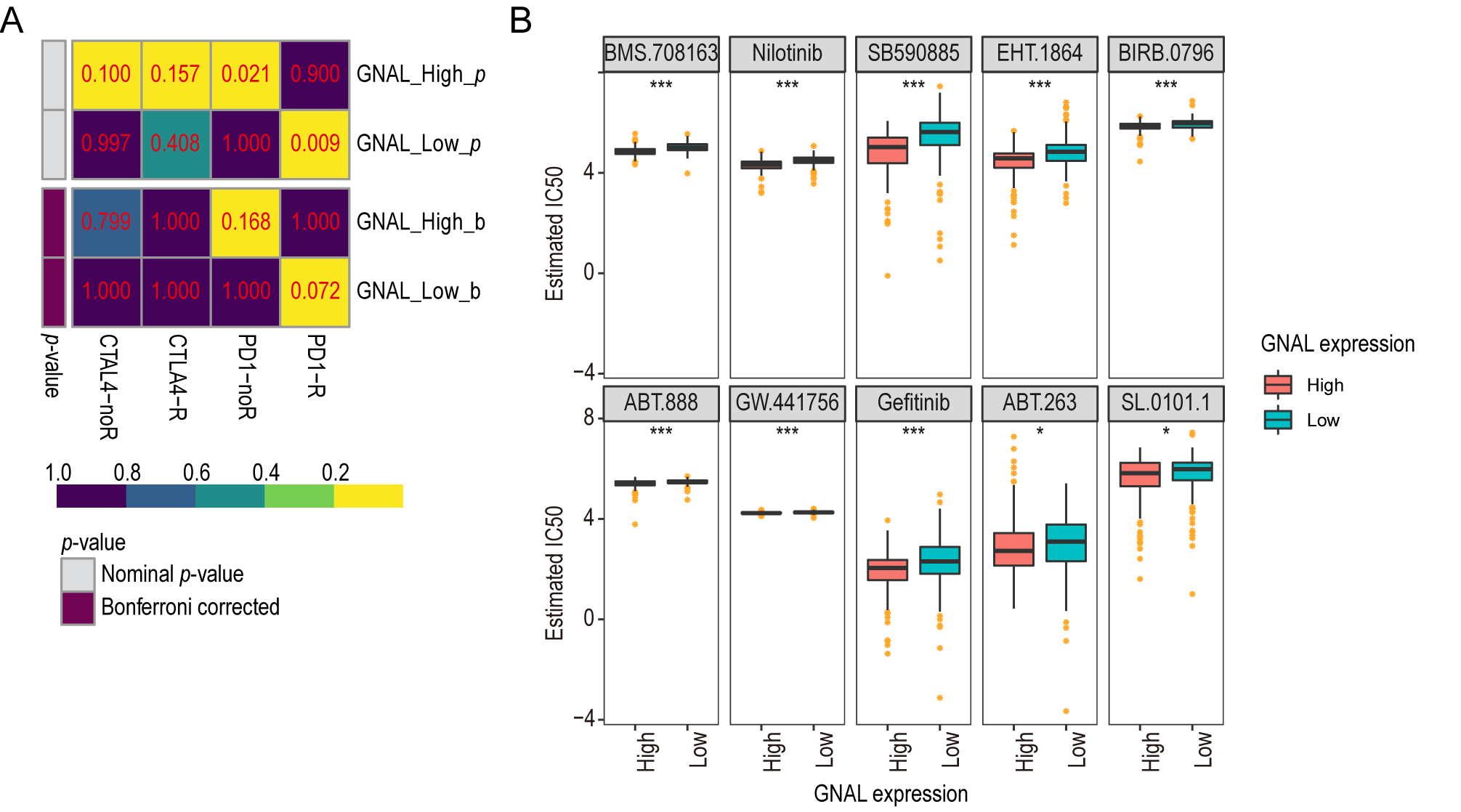

Supplement: Supplementary Fig. 9 [file OncolRes-32-45769-s009.tif]

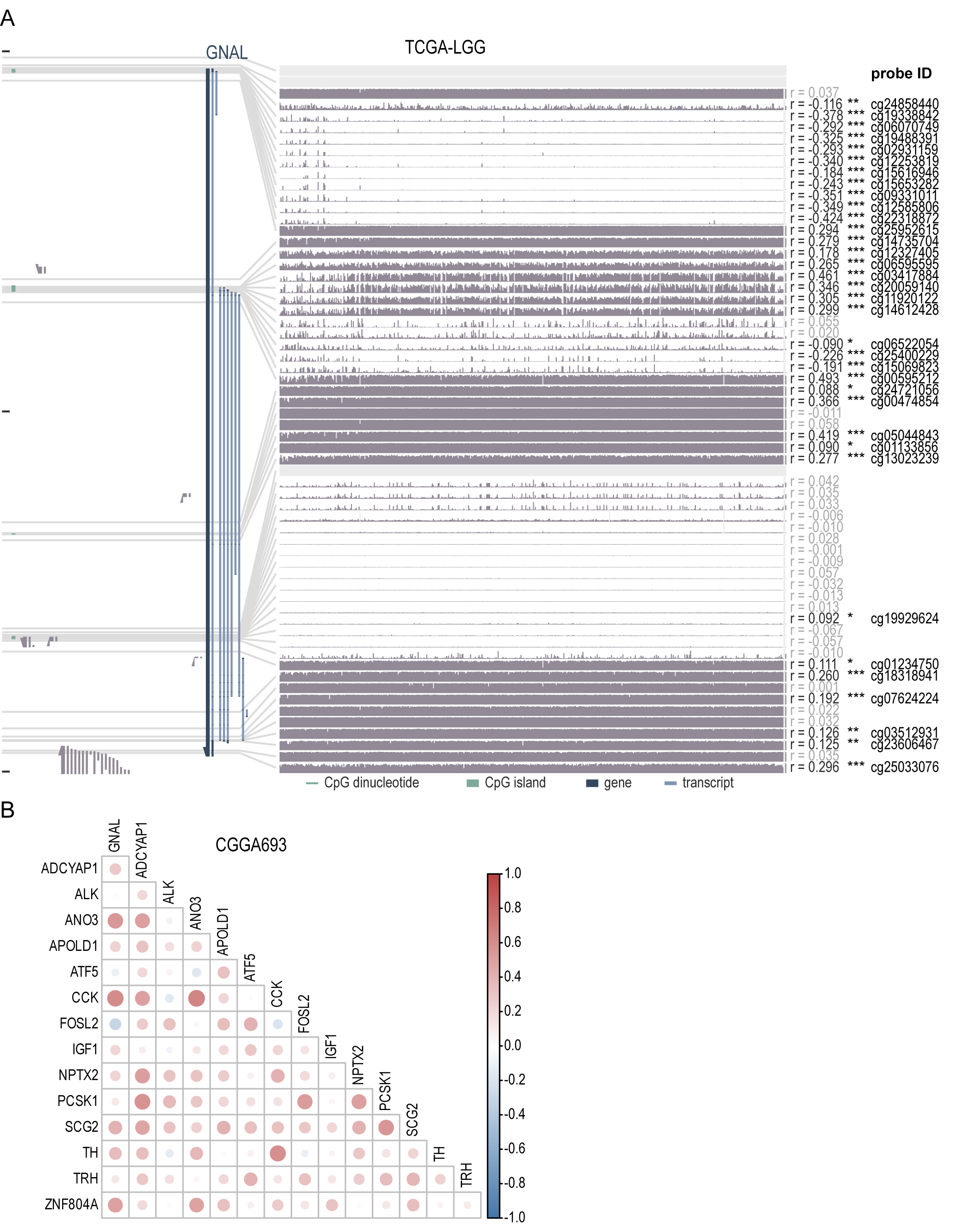

Supplement: Supplementary Fig. 10 [file OncolRes-32-45769-s010.tif]
