# Supplementary material for: Multi-cohort comprehensive analysis unveiling the clinical value and therapeutic effect of GNAL in glioma
Source: Oncol Res. 2024 Apr 23;32(5):965–81. doi: 10.32604/or.2024.045769 (PMC11055992; doi:10.32604/or.2024.045769)
Supplement: Supplementary file 11 [file OncolRes-32-45769-s001.docx]

**Table S1**

The detailed distribution of information for cohorts.

|  | CGGA325 | CGGA693 | TCGA-GBMLGG |
| --- | --- | --- | --- |
| Samples | 222 | 404 | 603 |
| Type of cancer |  |  |  |
| astrocytoma | 62 | 131 | 222 |
| oligodendrocytoma | 49 | 66 | 149 |
| glioblastoma | 109 | 170 | 139 |
| Gender |  |  |  |
| Female | 84 | 173 | 254 |
| Male | 138 | 231 | 349 |
| Age |  |  |  |
| <42 | 94 | 178 | 246 |
| ≥42 | 128 | 226 | 357 |
| Mean±SD | 44.01±12.37 | 44.07±12.91 | 47.37±15.32 |
| Grade |  |  |  |
| WHO II | 90 | 130 | 213 |
| WHO III | 47 | 141 | 238 |
| WHO IV | 85 | 133 | 152 |
| *IDH*_mutation_status |  |  |  |
| Mutant | 112 | 197 | 373 |
| Wildtype | 109 | 170 | 224 |
| 1p/19q_codeletion_status |  |  |  |
| Codel | 50 | 85 | 149 |
| Non-codel | 169 | 262 | 449 |
| Deceased patients | 138 | 209 | 179 |
| Median follow-up time (day) | 1362 | 1933 | 1515 |

SD: standard deviation

**Table S2**

The list of 29 OSPRGs.

| Gene Symbol | Gene ID |
| --- | --- |
| *ADCY3* | 109 |
| *ANO2* | 57101 |
| *CNGA4* | 1262 |
| *CNGB1* | 1258 |
| *EBF1* | 1879 |
| *GNAL* | 2774 |
| *GNB1* | 2782 |
| *GNG13* | 51764 |
| *LDB1* | 8861 |
| *LHX2* | 9355 |
| *OR13A1* | 79290 |
| *OR13J1* | 392309 |
| *OR2A7* | 01427 |
| *OR2C1* | 4993 |
| *OR2H2* | 7932 |
| *OR2L13* | 284521 |
| *OR2L2* | 26246 |
| *OR2W3* | 343171 |
| *OR4N2* | 390429 |
| *OR51E1* | 143503 |
| *OR51E2* | 81285 |
| *OR52N4* | 390072 |
| *OR5K2* | 402135 |
| *OR7A5* | 26659 |
| *OR7C1* | 26664 |
| *OR7D2* | 162998 |
| *OR8S1* | 341568 |
| *REEP1* | 65055 |
| *RTP1* | 132112 |

**Table S3**

Characteristics of patients between *GNAL* high and low-expression group in CGGA693 cohort.

| Characteristics | N | Low expression (N=202) | High expression (N=202) | *p*-value |
| --- | --- | --- | --- | --- |
| Grade | 404 |  |  | <0.001 |
| WHO II |  | 39(9.65%) | 91(22.52%) |  |
| WHO III |  | 57(14.11%) | 84(20.79%) |  |
| WHO IV |  | 106(26.24%) | 27(6.68%) |  |
| Gender | 404 |  |  | 0.07 |
| Female |  | 77(19.06%) | 96(23.76%) |  |
| Male |  | 125(30.94%) | 106(26.24%) |  |
| Age | 404 |  |  | <0.001 |
| <42 |  | 72(17.82%) | 107(26.49%) |  |
| ≥42 |  | 130(32.18%) | 95(23.51%) |  |
| *IDH* mutation status | 367 |  |  | <0.001 |
| Mutant |  | 72(19.62%) | 125(34.06%) |  |
| Wildtype |  | 123(33.51%) | 47(12.81%) |  |
| 1p/19q codeletion status | 347 |  |  | <0.001 |
| Codeletion |  | 5(1.44%) | 80(23.05%) |  |
| Non-codeletion |  | 143(41.21%) | 119(34.29%) |  |
| MGMTp methylation status | 333 |  |  | 0.975 |
| Methylated |  | 100(30.03%) | 90(27.03%) |  |
| Un-methylated |  | 75(22.52%) | 68(20.42%) |  |

**Table S4**

Characteristics of patients between *GNAL* high and low-expression group in TCGA-GBMLGG cohort.

| Characteristics | N | Low expression (N=302) | High expression (N=301) | *p-*value |
| --- | --- | --- | --- | --- |
| Grade | 603 |  |  | <0.001 |
| WHO II |  | 56(9.29%) | 157(26.04%) |  |
| WHO III |  | 97(16.09%) | 141(23.38%) |  |
| WHO IV |  | 149(24.71%) | 3(0.50%) |  |
| Gender | 603 |  |  | 0.54 |
| Female |  | 123(20.40%) | 131(21.72%) |  |
| Male |  | 179(29.68%) | 170(28.19%) |  |
| Age | 603 |  |  | <0.001 |
| <42 |  | 70(11.61%) | 176(29.19%) |  |
| ≥42 |  | 232(38.47%) | 125(20.73%) |  |
| *IDH* mutation status | 597 |  |  | <0.001 |
| Mutant |  | 101(16.92%) | 272(45.56%) |  |
| Wildtype |  | 198(33.17%) | 26(4.36%) |  |
| 1p/19q codeletion status | 598 |  |  | <0.001 |
| Codeletion |  | 14(2.34%) | 135(22.58%) |  |
| Non-codeletion |  | 283(47.32%) | 166(27.76%) |  |

**Table S5**

Univariate Cox regression analysis of clinicopathologic characteristics in public cohorts.

| Cohort | Clinicopathological  characteristics | Coefficient | *p-*value | Hazard ratio(95% CI) |
| --- | --- | --- | --- | --- |
| CGGA325 | Grade | 1.678 | <0.001 | 5.355(3.736-7.676) |
|  | Gender | 0.043 | 0.806 | 1.044(0.742-1.470) |
|  | Age | 0.052 | <0.001 | 1.054(1.038-1.070) |
|  | *IDH* mutation status | -1.535 | <0.001 | 0.216(0.150-0.310) |
|  | 1p/19q codeletion status | -2.228 | <0.001 | 0.108(0.054-0.213) |
|  | MGMTp methylation status | -0.461 | 0.009 | 0.631(0.446-0.893) |
| CGGA693 | Grade | 1.622 | <0.001 | 5.061(3.818-6.708) |
|  | Gender | -0.144 | 0.309 | 0.866(0.656-1.143) |
|  | Age | 0.040 | <0.001 | 1.041(1.029-1.053) |
|  | *IDH* mutation status | -1.486 | <0.001 | 0.226(0.168-0.305) |
|  | 1p/19q codeletion status | -1.788 | <0.001 | 0.167(0.097-0.289) |
|  | MGMTp methylation status | -0.305 | 0.043 | 0.737(0.548-0.991) |
| TCGA-GMBLGG | Grade | 2.259 | <0.001 | 9.576(6.835-13.42) |
|  | Gender | -0.001 | 0.997 | 0.999(0.742-1.345) |
|  | Age | 0.073 | <0.001 | 1.075(1.063-1.088) |
|  | *IDH* mutation status | -2.404 | <0.001 | 0.090(0.063-0.129) |
|  | 1p/19q codeletion status | -1.513 | <0.001 | 0.220(0.129-0.374) |

95% CI: 95% confidence intervals

**Table S6**

The correlation coefficients and *p-*values between *GNAL* and immune-related genes in public cohorts.

|  | CGGA325 | | CGGA693 | | TCGA-GBMLGG | |
| --- | --- | --- | --- | --- | --- | --- |
| genes | Coef | *p* | Coef | *p* | Coef | *p* |
| *PCSK2* | 0.730 | <0.0001 | 0.741 | <0.0001 | 0.647 | <0.0001 |
| *SLIT1* | 0.680 | <0.0001 | 0.620 | <0.0001 | 0.621 | <0.0001 |
| *FGF13* | 0.819 | <0.0001 | 0.793 | <0.0001 | 0.737 | <0.0001 |
| *GNAI1* | 0.660 | <0.0001 | 0.620 | <0.0001 | 0.640 | <0.0001 |
| *PRKCB* | 0.650 | <0.0001 | 0.714 | <0.0001 | 0.733 | <0.0001 |
| *PAK3* | 0.759 | <0.0001 | 0.602 | <0.0001 | 0.743 | <0.0001 |
| *NRG3* | 0.749 | <0.0001 | 0.656 | <0.0001 | 0.629 | <0.0001 |
| *CHGB* | 0.837 | <0.0001 | 0.828 | <0.0001 | 0.780 | <0.0001 |
| *CRHR1* | 0.686 | <0.0001 | 0.707 | <0.0001 | 0.688 | <0.0001 |
| *CHGA* | 0.792 | <0.0001 | 0.727 | <0.0001 | 0.695 | <0.0001 |
| *SSTR1* | 0.770 | <0.0001 | 0.705 | <0.0001 | 0.738 | <0.0001 |
| *PPP3CB* | 0.750 | <0.0001 | 0.714 | <0.0001 | 0.745 | <0.0001 |
| *SEMA4A* | 0.643 | <0.0001 | 0.684 | <0.0001 | 0.606 | <0.0001 |
| *SSTR2* | 0.723 | <0.0001 | 0.642 | <0.0001 | 0.701 | <0.0001 |
| *ARRB1* | 0.628 | <0.0001 | 0.640 | <0.0001 | 0.618 | <0.0001 |

Coef: Spearman’s correlation coefficient
